# Research on boundary slip of hydrostatic lead screw under different driving modes

**DOI:** 10.1038/s41598-021-01524-8

**Published:** 2021-11-16

**Authors:** Yandong Liu, Xianying Feng, Yanfei Li, Jiajia Lu, Zhe Su

**Affiliations:** 1 School of Mechanical Engineering, Shandong University, Jinan, 250061 Shandong China; 2 Key Laboratory of High Efficiency and Clean Mechanical Manufacture of Ministry of Education, Shandong University, Jinan, 250061 Shandong China

**Keywords:** Mechanical engineering, Mathematics and computing

## Abstract

The flow state of oil film in the hydrostatic lead screw directly affects the transmission performance of the screw pair. The static and dynamic characteristics of a new type of double driven hydrostatic screw-nut pair (DDHSNP) are studied under different motion modes. The boundary condition of navier slip model is introduced into the lubricating mathematical model of DDHSNP, and the influences of boundary slip on the axial bearing capacity, axial stiffness and damping coefficient in micro scale are researched by finite difference method. The results show that when the motor runs at high speed (the rotating speed range of the screw and nut driven motor is 1000–9000 rpm), the existence of boundary slip leads to a improvement of the axial bearing capacity and stiffness coefficient of DDHSNP in the case of single-drive operation and dual-drive differential feed (the range of rotation difference is 10–100 rpm), which is more obvious under the single-drive mode. The increase rate of stiffness coefficient induced by boundary slip is much larger than that of bearing capacity. In addition, the boundary slip has little effect on the damping coefficient of DDHSNP in either single drive operation or dual drive differential operation.

## Introduction

The machining accuracy of large stroke is greatly affected by the stability of the ultra-precision machine tools under the condition of a low speed. Therefore, how to achieve a high-precision control of the micro-displacement under ultra-low speed is extremely important^1^. A micro-nano motion method based on the principle of "differential" synthesis as well as ball screw pair screw transmission has been proposed before. This method avoids the crawling interval of the electromechanical system, long and precise micro-feed can be achieved^2–4^. Unfortunately, it is found that the theoretical nanometer precision can not be fully realized due to the unevenness of the ball under the tiny velocity difference, the geometric error of the spiral raceway and the surface roughness of the raceway.

The hydrostatic lead screw, transmits power by the oil films with high rigidity between screw threads and nut threads. Compared with traditional ball screws, the hydrostatic lead screw has many desirable characteristics such as high motion accuracy, high stiffness, frictionless running and high load capacity. In recent years, hydrostatic lead screw has been extensively applied in ultra-precision machine tools^5–7^. On the basis of our previous research^2^, a novel double driven hydrostatic screw-nut pair (DDHSNP) is designed based on the principle of relativity, hydrostatic lead screw transmission principle and "differential synthesis" principle to achieve higher motion accuracy. In addition to satisfying the general function of the hydrostatic lead screw transmission, the DDHSNP can not only drive the screw and nut respectively, but also achieve high-precision nanoscale motion through the differential synthesis of these two parts.

Many pioneering works on hydrostatic lead screw have been reported. In order to calculate the static.

characteristics of the hydrostatic lead screw precisely, El-Sayed and Khatan proposed an equivalent model to facilitate the calculation of the flank surface of threads and deduced a formula about the characteristics of hydrostatic lead screw. After that, El-Sayed and Khatan investigated the optimal parameters of trapezoidal and rectangular threads to get the largest load capacity and stiffness under constant power^8^. Moreover, they concluded that the performance of trapezoidal thread was better than that of rectangular thread by comparing the operating characteristics of the hydrostatic lead screw^9^. The transmission error of the hydrostatic lead screw can be smaller than pitch error because of the error homogenization of oil films. Based on the work of El-Sayed and Khatan, Zhang et al. researched the factors which influenced the error homogenization of oil films under low speed and constant external load^10,11^. In addition, Zhang et al. proposed two methods to improve the axial static and dynamic characteristics of the hydrostatic lead screw, including the use of membrane restrictor and formation of fluctuant film clearance by machining deliberate periodic pitch errors in the nut^12^.

Most of the recent studies are devoted to analyzing the structure and performance of nut, few reported the effect of boundary slip for the hydrostatic lead screw in the micro scale^8–12^. With the development of large-scale and high-speed mechanical equipment, the application of the hydrostatic lead screw faces the challenges of extreme working conditions such as high speeds and large loads. Liquid clearance flow differs from the macro-scale fluid flow behavior under extreme conditions. Due to the oil film thickness, high shear rate, and viscosity variation, oil film clearance flow is prone to exhibit viscoplastic behavior similar to solids, resulting in boundary slip at the solid–liquid interface^13,14^. Neglecting the boundary slip will seriously effect the calculation accuracy of the hydrodynamic parameters such as oil chamber flow, stiffness and load capacity^15^. Furthermore, the slip of the clearance oil film at the limit shear force can make the oil film yield and lose efficacy, eventually influences the dynamic characteristics of the hydrostatic lead screw^16^.

Boundary slip has fascinated much interest of researchers for decades. To acquire the dynamic characteristics of the hydrostatic spindle more comprehensively, Chen et al. researched the influence of oil film slip on load capacity and stiffness in spindle system. They confirmed the existence of slip in the process of oil film flow by measuring the axial stiffness of the spindle^17^. Aurelian et al. indicated that wall slip can better improve power loss than textured bearings. In addition, they found that the load capacity can be improved by both textured bearings and wall slip, but textured bearings only work in specific surface patterns^18^. Zhao et al. investigated the influence of the slip/no-slip on 2D elastohydrodynamic lubrication (EHL) and evaluated the effect of boundary slip on bearing performance in terms of oil film thickness and friction coefficient^19^. Although the majority of scholars have conducted numerous studies on boundary slip, there are few journal articles reporting the characteristics of the hydrostatic lead screw. By introducing the boundary slip theory into the calculation of oil film characteristics of the hydrostatic lead screw, the calculation will be further improved. It also provides a new perspective for the phenomena that cannot be explained by classical theories in the practical application of hydrostatic lead screw. Especially for DDHSNP, a new type of micro-nano feed technology, it is of great significance to study the influence of boundary slip on the performance of the DDHSNP under different transmission modes.

This paper researches the boundary slip phenomenon of hydrostatic lead screw under different driving modes. The remainder of this article is organized as follows. The structure of the DDHSNP is described in “The structure of DDHSNP” section. The mathematical equation of oil film characteristics in DDHSNP is given based on the conventional hydrostatic lead screw theory, the navier boundary slip model reflecting the micro scale size effect is introduced, and the reynolds equation is modified to make it more realistic in “Mathematical model of spiral oil film characteristics with boundary slip” section. The effect of boundary slip on the static and dynamic characteristics of DDHSNP under single-drive operation mode and dual-drive differential operation mode are investigated in “Oil film static and dynamic characteristics control equations with boundary slip” section and “Simulation results and analysis of static and dynamic characteristics with boundary slip” section, respectively. Finally, this study is concluded in “Conclusion” section.

## The structure of DDHSNP

**Figure 1:**
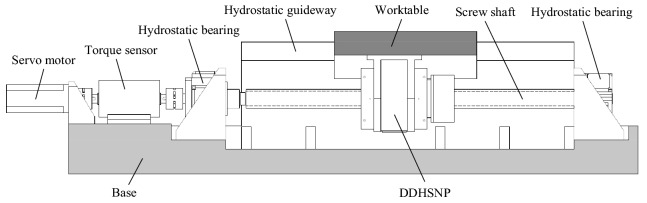
The micro-nano motion system based on DDHSNP.

In order to achieve high-precision and large-stroke motion control in nano-scale, a novel micro-nano motion method is proposed with the aid of the innovatively designed DDHSNP^20^. The mechanism diagram of the new micro-nano transmission system based on DDHSNP is shown in Fig. 1.

**Figure 2:**
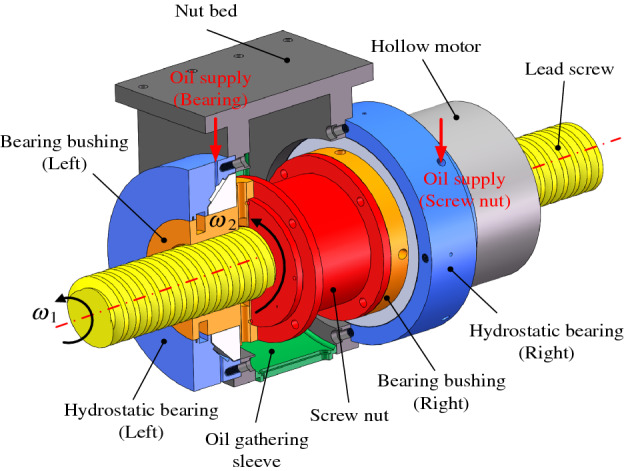
The schematic diagram of the DDHSNP structure.

As depicted in Fig. 2, with DDHSNP, not only can the conventional functions of hydrostatic lead screw be met, but also the motion in micro-nano scale can be realized through "differential synthesis" by driving the lead screw and nut with the same steering and nearly equal rotation speed. Both sides of the nut are equipped with bearing bushes and bearings to provide rotation pair for the screw nut. The nut is connected with the hollow motor rotor through the bearing bush to transmit power.

**Figure 3:**
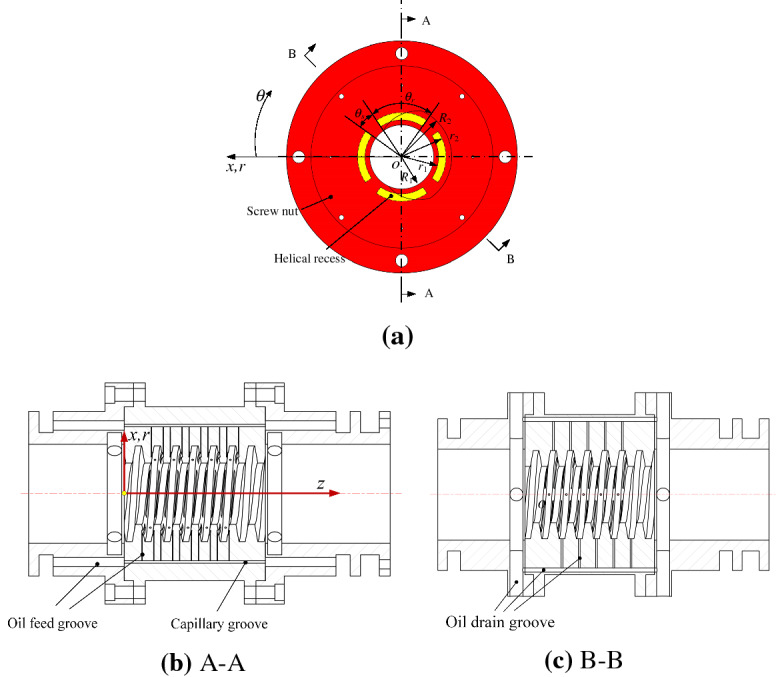
Internal structure and oil circuit of nut (**a**) Layout of the helical recesses, (**b**) A-A cross-section of the hydrostatic nut, (**c**) B-B cross-section of the hydrostatic nut.

Figure 3a–c show the internal structures of a conventional hydrostatic screw nut with capillary throttling. Four spiral grooves are evenly arranged on each side of the single thread lead length, and each spiral groove corresponds to a capillary restrictor. In particular, in order to simplify the manufacturing process, the screw nut and the bearing bush are both processed with oil inlet and outlet holes. After the screw nut and the bearing bush are matched, a complete oil circuit is formed to supply oil for the nut. As shown in Fig. 3b, the coordinate system is located on the screw nut, and the axis *oz* represents the center axis of the nut.

Assuming that the nut displacement between the actual and the ideal position of the nut along the positive direction of *z*-axis is Δ*h*, the oil film thickness on the left and right sides of the nut thread can be expressed as

1 $$\left\{ {\begin{array}{*{20}l} {h_{L}^{{\prime }} = h_{0} + \vartriangle h} \hfill \\ {h_{R}^{{\prime }} = h_{0} - \vartriangle h} \hfill \\ \end{array} } \right.$$

## Mathematical model of spiral oil film characteristics with boundary slip

### The establishment of equivalent model for spiral oil film

It can be seen from Ref.^21^ that the helical surface of the thread can be approximately replaced by the unfolded sectorial plane to obtain an equivalent model of the spiral oil film. The geometric relation between the spiral surface of the thread and the expanded setorial plane is as follows^21^:

2 $$R_{1}^{{\prime }} { = }R_{1} K_{{1}} , \, R_{2}^{{\prime }} { = }R_{{2}} K_{{2}}, \quad \theta_{\psi } = 2\pi K,r^{{\prime }} = r + e$$

where *K*, *K*_1_, *K*_2_ and *e* can be obtained by the Eq. (3):

3 $$\left\{ {\begin{array}{*{20}l} {K = \left[ {{{\cos \alpha } \mathord{\left/ {\vphantom {{\cos \alpha } {\left( {1 - R_{1} /R_{2} } \right)}}} \right. \kern-\nulldelimiterspace} {\left( {1 - R_{1} /R_{2} } \right)}}} \right]\left[ {\sec \lambda_{2} - \sqrt {\left( {R_{1} /R_{2} } \right)^{2} + \tan^{2} \lambda_{2} } } \right]} \hfill \\ {K_{1} = {1 \mathord{\left/ {\vphantom {1 {\left( {K\cos \lambda_{1} } \right)}}} \right. \kern-\nulldelimiterspace} {\left( {K\cos \lambda_{1} } \right)}}} \hfill \\ {K_{2} = {1 \mathord{\left/ {\vphantom {1 {\left( {K\cos \lambda_{2} } \right)}}} \right. \kern-\nulldelimiterspace} {\left( {K\cos \lambda_{2} } \right)}}} \hfill \\ {e = \left( {{{R_{2} } \mathord{\left/ {\vphantom {{R_{2} } {\cos \alpha }}} \right. \kern-\nulldelimiterspace} {\cos \alpha }}} \right)\left[ {{{\left( {R_{2} - R_{1} } \right)} \mathord{\left/ {\vphantom {{\left( {R_{2} - R_{1} } \right)} {\left( {R_{2} - R_{1} \cos \lambda_{2} /\cos \lambda_{1} } \right) - 1}}} \right. \kern-\nulldelimiterspace} {\left( {R_{2} - R_{1} \cos \lambda_{2} /\cos \lambda_{1} } \right) - 1}}} \right]} \hfill \\ \end{array} } \right.$$

**Figure 4:**
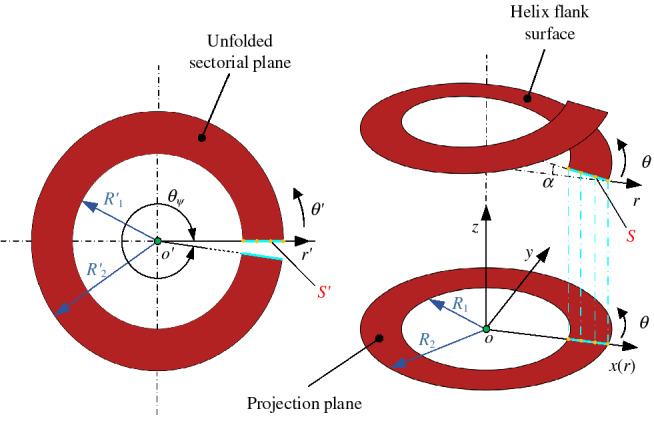
Equivalent drawing of the helix flank surface.

The coordinate system *o'*-*r'θ'* is located on the equivalent sectorial plane, as seen from Fig. 4. For any point *S* on the flank surface of threads, the corresponding position on the equivalent sectorial plane can be expressed as follows

4 $$S^{{\prime }} = \left[ {r^{{\prime }} \cos \theta^{{\prime }} ,r^{{\prime }} \sin \theta^{{\prime }} } \right]$$

where* r*′ is the radius of position *S*′ on the equivalent sectorial plane.

### The establishment of flow model for oil film

**Figure 5:**
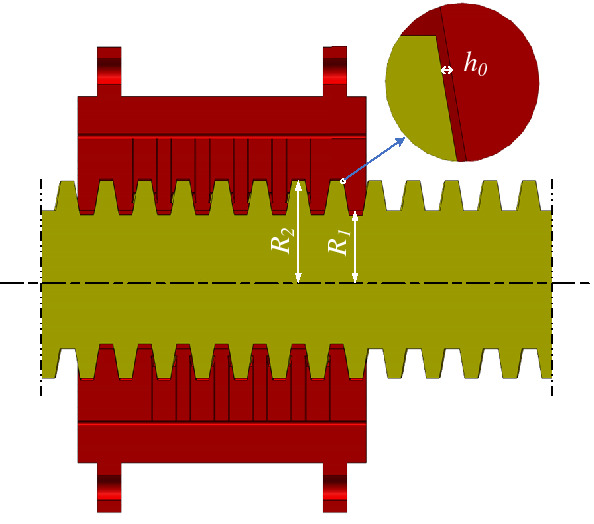
Schematic diagram of the axial engagement clearance of the hydrostatic lead screw.

Figure 5 shows the schematic diagram of the axial engagement clearance of the hydrostatic lead screw. It can be seen that the outer diameter *R*_2_ of the lead screw thread and the inner diameter *R*_1_ of the nut thread are both much larger than the thickness of clearance *h*_0_. The oil film flow is formed in the gap induced by the pressure difference as well as the rotation of the lead screw and nut. Since the clearance thickness *h*_0_ is only a few tens of microns, the oil film flow belongs to the category of micro-flow. For micron-level oil film clearance flow, the exact solution of the oil film equation varies with specific boundary conditions. Hence, it is of great significance to verify the slip boundary conditions which reflect the micro-scale effects.

Based on the theory of the equivalent sectorial plane of the helical surface, the flow of lubricant in the hydrostatic lead screw can be regarded as the flow of the clearance between two equivalent sectorial surfaces. In consideration of the general assumption of lubrication theory, the lubricating oil in the clearance is incompressible newtonian fluid and the continuous laminar flow. Assuming that the pressure on the spiral oil film along the thickness direction of the oil film remains constant, the flow control equations of the oil film in the coordinate system *o*′-*r*′*θ*′ are given by the following formula:

5 $$\left\{ {\begin{array}{*{20}l} {\frac{{\partial p^{{\prime }} }}{{r^{{\prime }} \partial \theta^{{\prime }} }} = \eta \frac{{\partial^{2} v_{{\theta^{{\prime }} }} }}{{\partial z^{{{\prime }2}} }}} \hfill \\ {\frac{{\partial p^{{\prime }} }}{{\partial r^{{\prime }} }} = \eta \frac{{\partial^{2} v_{{r^{{\prime }} }} }}{{\partial z^{{{\prime }2}} }} + \rho \frac{{v_{{\theta^{{\prime }} }}^{2} }}{{\rho_{r} }}\cos \alpha } \hfill \\ {\frac{{\partial p^{{\prime }} }}{{\partial z^{{\prime }} }} = 0} \hfill \\ \end{array} } \right.$$

where *p*′ is the pressure of the oil film, *v*_*θ'*_ is the circumferential velocity of oil film, *v*_*r'*_ is the radial speed of the oil film, *η* is the viscosity of the lubricating oil, *ρ* is the density of the lubricating oil, and *ρ*_*r*_ is the curvature radius of *r* at any radius.

### Boundary condition of boundary slip

**Figure 6:**
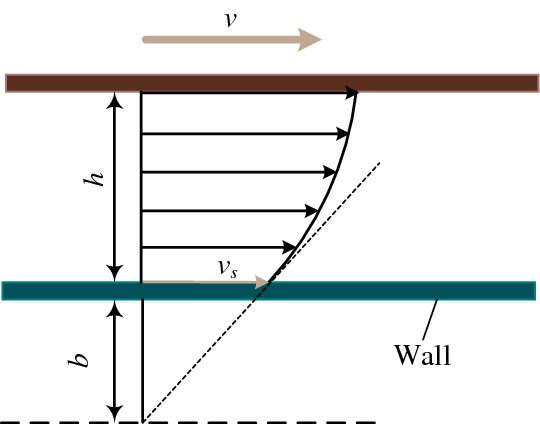
Schematic illustration of the navier slip model.

**Figure 7:**
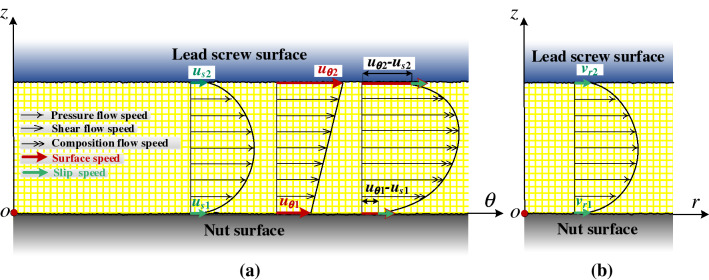
Oil film flow state of hydrostatic lead screw considering oil film slip (a) the circumferential direction (b) the radial direction.

In this paper, the navier slip model is used to research the oil film flow in the hydrostatic lead screw^14^. As shown in Fig. 6, assuming that the non-slip wall is located at the wall with a distance *b* from the actual solid-wall interface, and the slip speed is proportional to the local strain rate.

6 $$v_{s} = b\left( {\frac{\partial v}{{\partial z}}} \right)_{{{\text{Wall}}}}$$

where wall is the wall boundary, *b* is the oil film slip parameter, which reflects the material property, and its value is determined by experiment. As exhibited in Fig. 7, the reason why the circumferential flow of the oil film in the hydrostatic lead screw produces is that the pressure drives and the lead screw and nut rotates. The movement in the radial direction is caused by the pressure.

According to the navier slip model, the velocity boundary conditions of the oil film flow between the two flank surfaces of the hydrostatic lead screw are as follows:

7 $$\left\{ {\begin{array}{*{20}l} {\left. {z = 0\;u_{\theta 1} = u_{1} - u_{s1} = u_{1} - b_{\theta 1} \frac{\partial u}{{r\partial \theta }}} \right|_{0}\quad \left. {v_{r1} = b_{r1} \frac{\partial v}{{\partial r}}} \right|_{0} } \hfill \\ {\left. {z = h^{{\prime }} \;u_{\theta 2} = u_{2} - u_{s2} = u_{2} - b_{\theta 2} \frac{\partial u}{{r\partial \theta }}} \right|_{{h^{{\prime }} }}\quad \left. {v_{r2} = b_{r2} \frac{\partial v}{{\partial r}}} \right|_{{h^{{\prime }} }} } \hfill \\ \end{array} } \right.$$

where *u*_1_ and *u*_2_ are the rotational linear speeds of the nut and lead screw respectively, *u*_*s*1_ and *u*_*s*2_ are the slip speeds of the nut and lead screw surface in *θ'* direction respectively, *v*_*r*1_ and *v*_*r*2_ are the slip speeds of the nut and lead screw surface in *r'* direction respectively, *b*_*θ*1_ and *b*_*θ*2_ are the slip coefficients of the nut surface and the lead screw surface along the *θ'* direction, respectively, and *b*_*r*1_ and *b*_*r*2_ are the slip coefficients of the nut surface and the lead screw surface along the *r'* direction, respectively.

The velocity is decomposed according to the geometric relationship, and the axial velocity is set as *v*_*z'*_. For the right thread of the screw nut, the boundary values of *v*_*θ'*_, *v*_*r'*_, and *v*_*z'*_ are as follows:

8 $$\left\{ {\begin{array}{*{20}l} {v_{{\theta^{{\prime }} }} |_{{z^{{\prime }} = 0}} = \left( {\omega_{2} \sec^{2} \lambda_{r} - \omega_{1} \tan^{2} \lambda_{r} } \right)r\cos \lambda_{r} } \hfill \\ {v_{{\theta^{{\prime }} }} |_{{z^{{\prime }} = h^{{\prime }} }} = \omega_{1} r\cos \lambda_{r} } \hfill \\ {v_{{r^{{\prime }} }} |_{{z^{{\prime }} = 0}} = 0} \hfill \\ {v_{{r^{\prime}}} |_{{z^{{\prime }} = h^{{\prime }} }} = 0} \hfill \\ {v_{{z^{{\prime }} }} |_{{z^{{\prime }} = 0}} = \left[ { - \dot{z}_{nut} - \left( {{P \mathord{\left/ {\vphantom {P {2\pi }}} \right. \kern-\nulldelimiterspace} {2\pi }}} \right)\omega_{2} } \right]\cos \lambda_{r} \cos \alpha } \hfill \\ {v_{{z^{{\prime }} }} |_{{z^{{\prime }} = h^{{\prime }} }} = - \left( {{P \mathord{\left/ {\vphantom {P {2\pi }}} \right. \kern-\nulldelimiterspace} {2\pi }}} \right)\omega_{1} \cos \lambda_{r} \cos \alpha } \hfill \\ \end{array} } \right.$$

Substituting the oil film slip boundary condition Eq. (7) into Eq. (8), the velocity condition of oil film flow can be obtained by:

9 $$\left\{ \begin{aligned} & v_{{\theta ^{\prime}1}} |_{{z^{\prime} = 0}} {\text{ = }}(\omega _{2} \sec ^{2} \lambda _{r} - \omega _{1} \tan ^{2} \lambda _{r} )r\cos \lambda _{r} - {{b_{{\theta 1}} (\omega _{1} - \omega _{2} )r\cos \lambda _{r} \sec ^{2} \lambda _{r} } \mathord{\left/ {\vphantom {{b_{{\theta 1}} (\omega _{1} - \omega _{2} )r\cos \lambda _{r} \sec ^{2} \lambda _{r} } {\left( {b_{{\theta 2}} - b_{{\theta 1}} + h^{\prime}} \right)}}} \right. \kern-\nulldelimiterspace} {\left( {b_{{\theta 2}} - b_{{\theta 1}} + h^{\prime}} \right)}}{\text{ + }}\left( {{{b_{{\theta 1}} h^{\prime}} \mathord{\left/ {\vphantom {{b_{{\theta 1}} h^{\prime}} {2\eta }}} \right. \kern-\nulldelimiterspace} {2\eta }}} \right)\left[ {{{\left( {2b_{{\theta 2}} + h^{\prime}} \right)} \mathord{\left/ {\vphantom {{\left( {2b_{{\theta 2}} + h^{\prime}} \right)} {\left( {b_{{\theta 2}} - b_{{\theta 1}} + h^{\prime}} \right)}}} \right. \kern-\nulldelimiterspace} {\left( {b_{{\theta 2}} - b_{{\theta 1}} + h^{\prime}} \right)}}} \right]\frac{{\partial p^{\prime}}}{{r^{\prime}\partial \theta ^{\prime}}}{\text{ }} \\ & v_{{\theta ^{\prime}2}} |_{{z^{\prime} = h^{\prime}}} = \omega _{1} r\cos \lambda _{r} - \left( {{{b_{{\theta 2}} h^{\prime}} \mathord{\left/ {\vphantom {{b_{{\theta 2}} h^{\prime}} \eta }} \right. \kern-\nulldelimiterspace} \eta }} \right)\frac{{\partial p^{\prime}}}{{r^{\prime}\partial \theta ^{\prime}}} - {{b_{{\theta 2}} (\omega _{1} - \omega _{2} )r\cos \lambda _{r} \sec ^{2} \lambda _{r} } \mathord{\left/ {\vphantom {{b_{{\theta 2}} (\omega _{1} - \omega _{2} )r\cos \lambda _{r} \sec ^{2} \lambda _{r} } {\left( {b_{{\theta 2}} - b_{{\theta 1}} + h^{\prime}} \right){\text{ }}}}} \right. \kern-\nulldelimiterspace} {\left( {b_{{\theta 2}} - b_{{\theta 1}} + h^{\prime}} \right){\text{ }}}} + \left( {{{b_{{\theta 2}} h^{\prime}} \mathord{\left/ {\vphantom {{b_{{\theta 2}} h^{\prime}} {2\eta }}} \right. \kern-\nulldelimiterspace} {2\eta }}} \right)\left[ {{{\left( {2b_{{\theta 2}} + h^{\prime}} \right)} \mathord{\left/ {\vphantom {{\left( {2b_{{\theta 2}} + h^{\prime}} \right)} {\left( {b_{{\theta 2}} - b_{{\theta 1}} + h^{\prime}} \right)}}} \right. \kern-\nulldelimiterspace} {\left( {b_{{\theta 2}} - b_{{\theta 1}} + h^{\prime}} \right)}}} \right]\frac{{\partial p^{\prime}}}{{r^{\prime}\partial \theta ^{\prime}}} \\ & v_{{r^{\prime}1}} |_{{z^{\prime} = 0}} {\text{ = }}\left( {{{b_{{r1}} h^{\prime}} \mathord{\left/ {\vphantom {{b_{{r1}} h^{\prime}} {2\eta }}} \right. \kern-\nulldelimiterspace} {2\eta }}} \right)\left[ {{{\left( {h^{\prime} - 2b_{{r2}} } \right)} \mathord{\left/ {\vphantom {{\left( {h^{\prime} - 2b_{{r2}} } \right)} {\left( {b_{{r2}} - b_{{r1}} - h^{\prime}} \right)}}} \right. \kern-\nulldelimiterspace} {\left( {b_{{r2}} - b_{{r1}} - h^{\prime}} \right)}}} \right]\frac{{\partial p^{\prime}}}{{\partial r^{\prime}}} - \left( {{{b_{{r1}} \rho } \mathord{\left/ {\vphantom {{b_{{r1}} \rho } {2\eta }}} \right. \kern-\nulldelimiterspace} {2\eta }}} \right)\left( {{{h^{\prime}\cos \alpha } \mathord{\left/ {\vphantom {{h^{\prime}\cos \alpha } {\rho _{r} }}} \right. \kern-\nulldelimiterspace} {\rho _{r} }}} \right)\left[ {{{\left( {h^{\prime}\int_{0}^{{h^{\prime}}} {\int_{0}^{{h^{\prime}}} {v_{{\theta ^{\prime}}}^{2} dz^{\prime}dz^{\prime}} } - 2b_{{r2}} \int_{0}^{{h^{\prime}}} {v_{{\theta ^{\prime}}}^{2} dz^{\prime}} } \right)} \mathord{\left/ {\vphantom {{\left( {h^{\prime}\int_{0}^{{h^{\prime}}} {\int_{0}^{{h^{\prime}}} {v_{{\theta ^{\prime}}}^{2} dz^{\prime}dz^{\prime}} } - 2b_{{r2}} \int_{0}^{{h^{\prime}}} {v_{{\theta ^{\prime}}}^{2} dz^{\prime}} } \right)} {\left( {b_{{r2}} - b_{{r1}} - h^{\prime}} \right)}}} \right. \kern-\nulldelimiterspace} {\left( {b_{{r2}} - b_{{r1}} - h^{\prime}} \right)}}} \right] \\ & v_{{r^{\prime}2}} |_{{z^{\prime} = h^{\prime}}} = \left( {{{b_{{r2}} h^{\prime}} \mathord{\left/ {\vphantom {{b_{{r2}} h^{\prime}} \eta }} \right. \kern-\nulldelimiterspace} \eta }} \right)\frac{{\partial p^{\prime}}}{{\partial r^{\prime}}} - \left( {{{b_{{r2}} \rho \cos \alpha } \mathord{\left/ {\vphantom {{b_{{r2}} \rho \cos \alpha } \eta }} \right. \kern-\nulldelimiterspace} \eta }} \right)\left( {{{\int_{0}^{{h^{\prime}}} {v_{{\theta ^{\prime}}}^{2} dz^{\prime}} } \mathord{\left/ {\vphantom {{\int_{0}^{{h^{\prime}}} {v_{{\theta ^{\prime}}}^{2} dz^{\prime}} } {\rho _{r} }}} \right. \kern-\nulldelimiterspace} {\rho _{r} }}} \right) + \left( {{{b_{{r2}} h^{\prime}} \mathord{\left/ {\vphantom {{b_{{r2}} h^{\prime}} {2\eta }}} \right. \kern-\nulldelimiterspace} {2\eta }}} \right)\left[ {{{\left( {h^{\prime} - 2b_{{r2}} } \right)} \mathord{\left/ {\vphantom {{\left( {h^{\prime} - 2b_{{r2}} } \right)} {\left( {b_{{r2}} - b_{{r1}} - h^{\prime}} \right)}}} \right. \kern-\nulldelimiterspace} {\left( {b_{{r2}} - b_{{r1}} - h^{\prime}} \right)}}} \right]\frac{{\partial p^{\prime}}}{{\partial r^{\prime}}} - \left( {{{b_{{r2}} \rho } \mathord{\left/ {\vphantom {{b_{{r2}} \rho } {2\eta }}} \right. \kern-\nulldelimiterspace} {2\eta }}} \right)\left( {{{h^{\prime}\cos \alpha } \mathord{\left/ {\vphantom {{h^{\prime}\cos \alpha } {\rho _{r} }}} \right. \kern-\nulldelimiterspace} {\rho _{r} }}} \right)\left[ {{{\left( {h^{\prime}\int_{0}^{{h^{\prime}}} {\int_{0}^{{h^{\prime}}} {v_{{\theta ^{\prime}}}^{2} dz^{\prime}dz^{\prime}} } - 2b_{{r2}} \int_{0}^{{h^{\prime}}} {v_{{\theta ^{\prime}}}^{2} dz^{\prime}} } \right)} \mathord{\left/ {\vphantom {{\left( {h^{\prime}\int_{0}^{{h^{\prime}}} {\int_{0}^{{h^{\prime}}} {v_{{\theta ^{\prime}}}^{2} dz^{\prime}dz^{\prime}} } - 2b_{{r2}} \int_{0}^{{h^{\prime}}} {v_{{\theta ^{\prime}}}^{2} dz^{\prime}} } \right)} {\left( {b_{{r2}} - b_{{r1}} - h^{\prime}} \right)}}} \right. \kern-\nulldelimiterspace} {\left( {b_{{r2}} - b_{{r1}} - h^{\prime}} \right)}}} \right]{\text{ }} \\ \end{aligned} \right.$$

For isotropic liquid, the slip coefficient in different directions is equal, that is, *b*_*θ*1_ = *b*_*r*1_, *b*_*θ*2_ = *b*_*r*2_. In order to simplify the model, *b*_*θ*1_ = *b*_*r*1_ = *b*_*θ*2_ = *b*_*r*2_ = *b* is assumed. For the left boundary velocity, it is only necessary to exchange *v*|_*z'*=0_ and *v*|_*z'*=*h'*_ in Eq. (8).

### Modified reynolds equation by boundary slip

By integrating the oil film flow equation Eq. (5) with the boundary condition Eq. (9) of boundary slip, the velocity distribution of the oil film along the circumferential and radial directions can be given as follows:

10 $$\left\{ {\begin{array}{*{20}l} {v_{{\theta '}} = \frac{1}{{2\eta }}\frac{{\partial p^{{\prime }} }}{{r^{{\prime }} \partial \theta ^{{\prime }} }}z^{{{\prime 2}}} + \left[ {{{\left( {v_{{\theta ^{{{\prime 2}}} }} - v_{{\theta ^{{{\prime 1}}} }} } \right)} \mathord{\left/ {\vphantom {{\left( {v_{{\theta ^{{{\prime 2}}} }} - v_{{\theta ^{{{\prime 1}}} }} } \right)} h}} \right. \kern-\nulldelimiterspace} h}^{\prime } - \frac{1}{{2\eta }}\frac{{\partial p^{{\prime }} }}{{r^{{\prime }} \partial \theta ^{{\prime }} }}h^{{\prime }} } \right]z^{{\prime }} + v_{{\theta ^{{{\prime 1}}} }} } \hfill \\ \begin{gathered} v_{{r^{{\prime }} }} = \frac{1}{{2\eta }}\frac{{\partial p^{{\prime }} }}{{\partial r^{{\prime }} }}\left[ {z^{{{\prime 2}}} - h^{{\prime }} z^{{\prime }} } \right] - \frac{{\rho \cos \alpha }}{{\eta \rho _{r} }}\left\{ \begin{gathered} \left[ {\frac{{z^{{{\prime 6}}} }}{{120\eta ^{2} }} - \frac{{h^{{\prime }} z^{{{\prime 5}}} }}{{40\eta ^{2} }} + \frac{{h^{{{\prime 2}}} z^{{{\prime 4}}} }}{{48\eta ^{2} }}} \right]\left( {\frac{{\partial p^{{\prime }} }}{{r^{{\prime }} \partial \theta ^{{\prime }} }}} \right)^{2} \hfill \\ + \left[ {\frac{{\left( {v_{{\theta ^{{{\prime 2}}} }} - v_{{\theta ^{{{\prime 1}}} }} } \right)z^{{{\prime 5}}} }}{{20\eta h^{\prime}}} - \frac{{\left( {v_{{\theta ^{{{\prime 2}}} }} - 2v_{{\theta ^{{{\prime 1}}} }} } \right)z^{{{\prime 4}}} }}{{12\eta }} - \frac{{h^{{\prime }} v_{{\theta ^{{{\prime 1}}} }} z^{{{\prime 3}}} }}{{6\eta }}} \right]\left( {\frac{{\partial p^{{\prime }} }}{{r^{{\prime }} \partial \theta ^{{\prime }} }}} \right) \hfill \\ + \frac{{\left( {v_{{\theta ^{{{\prime 2}}} }} - v_{{\theta ^{{{\prime 1}}} }} } \right)^{2} z^{{{\prime 4}}} }}{{12h^{{{\prime 2}}} }} + \frac{{v_{{\theta ^{{{\prime 1}}} }} \left( {v_{{\theta ^{{{\prime 2}}} }} - v_{{\theta ^{{{\prime 1}}} }} } \right)z^{{{\prime 3}}} }}{{3h^{\prime}}} + \frac{{v_{{\theta ^{{{\prime 1}}} }}^{2} z^{{{\prime 2}}} }}{2} \hfill \\ - \frac{{z^{{\prime }} }}{{h^{{\prime }} }}\left[ {\frac{1}{{240\eta ^{2} }}\left( {\frac{{\partial p^{{\prime }} }}{{r^{{\prime }} \partial \theta ^{{\prime }} }}} \right)^{2} - \left( {\frac{{\left( {v_{{\theta ^{{{\prime 2}}} }} - v_{{\theta ^{{{\prime 1}}} }} } \right)h^{{{\prime 4}}} }}{{30\eta }} - \frac{1}{{12}}\frac{{v_{{\theta ^{{{\prime 1}}} }} }}{\eta }h^{{{\prime 4}}} } \right)\left( {\frac{{\partial p^{{\prime }} }}{{r^{{\prime }} \partial \theta ^{{\prime }} }}} \right) + \frac{{\left( {v_{{\theta ^{{{\prime 2}}} }}^{2} + 3v_{{\theta ^{{{\prime 1}}} }}^{2} + 2v_{{\theta ^{{{\prime 1}}} }} v_{{\theta ^{{{\prime 2}}} }} } \right)h^{{{\prime 2}}} }}{{12}}} \right] \hfill \\ \end{gathered} \right\} \hfill \\ + \frac{{v_{{r^{{\prime 2}} }} z^{{\prime }} }}{{h^{{\prime }} }} - \frac{{v_{{r^{{\prime 1}} }} \left( {z^{{\prime }} - h^{{\prime }} } \right)}}{{h^{{\prime }} }} \hfill \\ \end{gathered} \hfill \\ \end{array} } \right.$$

Integrating the Eq. (10) along the thickness direction of the oil film, the flow per unit oil film in *θ'* direction and *r'* direction can be expressed by

11 $$\left\{ \begin{aligned} & q_{{\theta^{\prime}}} = - \frac{{h^{{\prime}{3}} }}{12\eta }\frac{{\partial p^{\prime}}}{{r^{\prime}\partial \theta^{\prime}}} + \frac{{h^{\prime}}}{2}(v_{{\theta^{\prime}1}} + v_{{\theta^{\prime}2}} ) \\ & q_{{r^{\prime}}} = - \frac{{h^{{\prime}{3}} }}{12\eta }\frac{{\partial p^{\prime}}}{{\partial r^{\prime}}}{ + }\frac{{h^{\prime}(v_{{r^{\prime}1}} + v_{{r^{\prime}2}} )}}{2} - \frac{\rho \cos \alpha }{{\eta r_{c} }}\left[ \begin{gathered} - \frac{{h^{{\prime}{7}} }}{{1120\eta^{2} r^{2} }}\left( {\frac{{\partial p^{\prime}}}{{\partial \theta^{\prime}}}} \right)^{2} + \frac{{(v_{{\theta^{\prime}2}} - v_{{\theta^{\prime}1}} )h^{{\prime}{5}} }}{120\eta }\frac{{\partial p^{\prime}}}{{r\partial \theta^{\prime}}} \hfill \\ - \frac{{\left( {{3}v_{{\theta^{\prime}1}}^{2} + {3}v_{{\theta^{\prime}2}}^{2} + 4v_{{\theta^{\prime}1}} v_{{\theta^{\prime}2}} } \right)}}{120}h^{{\prime}{3}} \hfill \\ \end{gathered} \right] \, \\ \end{aligned} \right.$$

According to the assumption of incompressible lubrication and the flow conservation equation, Eq. (12) can be written as:

12 $$\frac{{\partial (q_{{\theta^{\prime}}} )}}{{\partial \theta^{\prime}}} + \frac{{\partial (r^{\prime}q_{{r^{\prime}}} )}}{{\partial r^{\prime}}} + r^{\prime}\frac{{\partial (q_{{z^{\prime}}} )}}{{\partial z^{\prime}}} = 0$$

Assuming that the rotation of the DDHSNP is in a steady state, the reynolds equation of the oil film slip can be derived by substituting Eq. (11) into Eq. (12) as follows:

13 $$\frac{\partial }{{\partial r^{\prime}}}\left( {r^{\prime}h^{{\prime}{3}} \frac{{\partial p^{\prime}}}{{\partial r^{\prime}}}} \right) + \frac{1}{{r^{\prime}}}\frac{\partial }{{\partial \theta^{\prime}}}\left( {h^{{\prime}{3}} \frac{{\partial p^{\prime}}}{{\partial \theta^{\prime}}}} \right) = 12\rho \cos \alpha \frac{\partial }{{\partial r^{\prime}}}\left( {\frac{{r^{\prime}h^{{\prime}{3}} }}{{r_{c} }}\frac{{V_{{\theta^{\prime}_{1} \theta^{\prime}_{2} }} }}{120}} \right) + 6\eta \frac{\partial }{{\partial r^{\prime}}}\left( {r^{\prime}h^{\prime}V_{{r^{\prime}}} } \right) + 6\eta V_{{\theta^{\prime}}} \frac{{\partial h^{\prime}}}{{\partial \theta^{\prime}}} \pm 12\eta r^{\prime}\left( {v_{{z^{\prime}2}} - v_{{z^{\prime}1}} } \right)$$

where

14 $$V_{{\theta^{\prime}_{1} \theta^{\prime}_{2} }} = \omega_{1}^{2} \left\{ \begin{gathered} 3\left[ {(\overline{\omega }\sec^{2} \lambda_{r} - \tan^{2} \lambda_{r} )r\cos \lambda_{r} - \frac{b}{{h^{\prime}}}(1 - \overline{\omega })r\cos \lambda_{r} \sec^{2} \lambda_{r} } \right]^{2} \hfill \\ + \left[ \begin{gathered} (\overline{\omega }\sec^{2} \lambda_{r} - \tan^{2} \lambda_{r} )r\cos \lambda_{r} - \hfill \\ \frac{b}{{h^{\prime}}}(1 - \overline{\omega })r\cos \lambda_{r} \sec^{2} \lambda_{r} \hfill \\ \end{gathered} \right]\left[ \begin{gathered} \frac{{10b^{2} + bh^{\prime}}}{{\eta \omega_{1} }}\frac{{\partial p^{\prime}}}{{r^{\prime}\partial \theta }} + 4r\cos \lambda_{r} \hfill \\ - \frac{4b}{{h^{\prime}}}(1 - \overline{\omega })r\cos \lambda_{r} \sec^{2} \lambda_{r} \hfill \\ \end{gathered} \right] \hfill \\ + 3\left[ \begin{gathered} r\cos \lambda_{r} \hfill \\ - \frac{b}{{h^{\prime}}}(1 - \overline{\omega })r\cos \lambda_{r} \sec^{2} \lambda_{r} \hfill \\ \end{gathered} \right]^{2} + \frac{{28b^{4} - b^{2} h^{{\prime}{2}} - 8b^{3} h^{\prime}}}{{4\eta^{2} \omega_{1}^{2} }}\left( {\frac{{\partial p^{\prime}}}{{r^{\prime}\partial \theta }}} \right)^{2} \hfill \\ + \left[ {r\cos \lambda_{r} - \frac{b}{{h^{\prime}}}(1 - \overline{\omega })r\cos \lambda_{r} \sec^{2} \lambda_{r} } \right]\frac{{10b^{2} - bh^{\prime}}}{{\eta \omega_{1} }}\frac{{\partial p^{\prime}}}{{r^{\prime}\partial \theta }} \hfill \\ \end{gathered} \right\}$$

15 $$\begin{aligned} V_{{r^{\prime}}} = & \frac{{2b^{2} }}{\eta }\frac{{\partial p^{\prime}}}{{\partial r^{\prime}}} \\ & \; + \frac{b\rho \cos \alpha }{{\eta r_{C} }}\left\{ \begin{gathered} \frac{{h^{{\prime}{7}} }}{{240\eta^{2} }}\left( {\frac{{\partial p^{\prime}}}{{r^{\prime}\partial \theta }}} \right)^{2} - \frac{{h^{{\prime}{5}} (3v_{{\theta^{\prime}1}} + 2v_{{\theta^{\prime}2}} )}}{60\eta }\frac{{\partial p^{\prime}}}{{r^{\prime}\partial \theta }} + \frac{{3v_{{\theta^{\prime}1}}^{2} + v_{{\theta^{\prime}2}}^{2} + 2v_{{\theta^{\prime}1}} v_{{\theta^{\prime}2}} }}{12} \hfill \\ - (2b + 1)\left[ \begin{gathered} \frac{{h^{{\prime}{5}} }}{{120\eta^{2} }}\left( {\frac{{\partial p^{\prime}}}{{r^{\prime}\partial \theta }}} \right)^{2} - \frac{{h^{{\prime}{3}} (v_{{\theta^{\prime}1}} + v_{{\theta^{\prime}2}} )}}{12\eta }\frac{{\partial p^{\prime}}}{{r^{\prime}\partial \theta }} \hfill \\ + \frac{{h^{\prime}(v_{{\theta^{\prime}2}}^{2} + v_{{\theta^{\prime}1}}^{2} + v_{{\theta^{\prime}1}} v_{{\theta^{\prime}2}} )}}{3} \hfill \\ \end{gathered} \right] \hfill \\ \end{gathered} \right\} \\ \end{aligned}$$

16 $$V_{{\theta^{\prime}}} = \omega_{1} \left[ {\overline{\omega }\sec^{2} \lambda_{r} - \tan^{2} \lambda_{r} + 1 - \frac{2b}{{h^{\prime}}}(1 - \overline{\omega })\sec^{2} \lambda_{r} } \right]r\cos \lambda_{r} + \frac{{2b^{2} }}{\eta }\frac{{\partial p^{\prime}}}{{r^{\prime}\partial \theta }}$$

Since *h* <  < *r′*, the Eq. (15) can be simplified to

17 $$V_{{r^{\prime}}} = \frac{{2b^{2} }}{\eta }\frac{{\partial p^{\prime}}}{{\partial r^{\prime}}} + \frac{b\rho }{{12\eta }}\frac{\cos \alpha }{{r_{C} }}\left\{ \begin{gathered} (3h^{{\prime}{3}} - 8bh^{\prime} - 4h^{\prime})v_{{\theta^{\prime}1}}^{2} + (h^{{\prime}{3}} - 8bh^{\prime} - 4h^{\prime})v_{{\theta^{\prime}2}}^{2} \hfill \\ + (2h^{{\prime}{3}} - 8bh^{\prime} - 4h^{\prime})v_{{\theta^{\prime}1}} v_{{\theta^{\prime}2}} + \frac{{h^{{\prime}{3}} (2b + 1)}}{\eta }(v_{{\theta^{\prime}1}} + v_{{\theta^{\prime}2}} )\frac{{\partial p^{\prime}}}{{r^{\prime}\partial \theta }} \hfill \\ \end{gathered} \right\}$$

The *V*_*r'*_ can be derived by substituting *v*_*θ'*1_ and *v*_*θ'*2_ into Eq. (17), where $\overline{\omega } = {{\omega_{2} } \mathord{\left/ {\vphantom {{\omega_{2} } {\omega_{1} }}} \right. \kern-\nulldelimiterspace} {\omega_{1} }}$.

18 $$\begin{gathered} V_{{r^{\prime}}} = \frac{{2b^{2} }}{\eta }\frac{{\partial p^{\prime}}}{{\partial r^{\prime}}} \hfill \\ + \frac{b\rho \cos \alpha }{{\eta r_{C} }}\left\{ \begin{gathered} \frac{{\left( {3h^{{\prime}{3}} - 8bh^{\prime} - 4h^{\prime}} \right)\omega_{1}^{2} }}{12}\left\{ \begin{gathered} \left[ \begin{gathered} (\overline{\omega }\sec^{2} \lambda_{r} - \tan^{2} \lambda_{r} )r\cos \lambda_{r} \hfill \\ - \frac{b}{{h^{\prime}}}(1 - \overline{\omega })r\cos \lambda_{r} \sec^{2} \lambda_{r} \hfill \\ \end{gathered} \right]^{2} \hfill \\ + \frac{{(2b^{2} + bh^{\prime})^{2} }}{{4\eta^{2} \omega_{1}^{2} }}\left( {\frac{{\partial p^{\prime}}}{{r^{\prime}\partial \theta }}} \right)^{2} \hfill \\ + 2\left[ \begin{gathered} (\overline{\omega }\sec^{2} \lambda_{r} - \tan^{2} \lambda_{r} )r\cos \lambda_{r} \hfill \\ - \frac{b}{{h^{\prime}}}(1 - \overline{\omega })r\cos \lambda_{r} \sec^{2} \lambda_{r} \hfill \\ \end{gathered} \right]\frac{{2b^{2} + bh^{\prime}}}{{2\eta \omega_{1} }}\frac{{\partial p^{\prime}}}{{r^{\prime}\partial \theta }} \hfill \\ \end{gathered} \right\} \hfill \\ + \frac{{\left( {h^{{\prime}{3}} - 8bh^{\prime} - 4h^{\prime}} \right)\omega_{1}^{2} }}{12}\left\{ \begin{gathered} \left[ {r\cos \lambda_{r} - \frac{b}{{h^{\prime}}}(1 - \overline{\omega })r\cos \lambda_{r} \sec^{2} \lambda_{r} } \right]^{2} \hfill \\ + \frac{{(2b^{2} - bh^{\prime})^{2} }}{{4\eta^{2} \omega_{1}^{2} }}\left( {\frac{{\partial p^{\prime}}}{{r^{\prime}\partial \theta }}} \right)^{2} \hfill \\ + 2\left[ {r\cos \lambda_{r} - \frac{b}{{h^{\prime}}}(1 - \overline{\omega })r\cos \lambda_{r} \sec^{2} \lambda_{r} } \right]\frac{{2b^{2} - bh^{\prime}}}{{2\eta \omega_{1} }}\frac{{\partial p^{\prime}}}{{r^{\prime}\partial \theta }} \hfill \\ \end{gathered} \right\} \hfill \\ + \frac{{\left( {2h^{{\prime}{3}} - 8bh^{\prime} - 4h^{\prime}} \right)\omega_{1}^{2} }}{12}\left\{ \begin{gathered} \left[ \begin{gathered} (\overline{\omega }\sec^{2} \lambda_{r} - \tan^{2} \lambda_{r} )r\cos \lambda_{r} \hfill \\ - \frac{b}{{h^{\prime}}}(1 - \overline{\omega })r\cos \lambda_{r} \sec^{2} \lambda_{r} \hfill \\ \end{gathered} \right]\left[ \begin{gathered} r\cos \lambda_{r} - \frac{{bh^{\prime}}}{{\eta \omega_{1} }}\frac{{\partial p^{\prime}}}{{r^{\prime}\partial \theta }} \hfill \\ - \frac{b}{{h^{\prime}}}(1 - \overline{\omega })r\cos \lambda_{r} \sec^{2} \lambda_{r} \hfill \\ \end{gathered} \right] \hfill \\ + \left[ \begin{gathered} (\overline{\omega }\sec^{2} \lambda_{r} - \tan^{2} \lambda_{r} )r\cos \lambda_{r} \hfill \\ - \frac{b}{{h^{\prime}}}(1 - \overline{\omega })r\cos \lambda_{r} \sec^{2} \lambda_{r} \hfill \\ \end{gathered} \right]\frac{{2b^{2} + bh^{\prime}}}{{2\eta \omega_{1} }}\frac{{\partial p^{\prime}}}{{r^{\prime}\partial \theta }} \hfill \\ + \left[ \begin{gathered} r\cos \lambda_{r} - \frac{{bh^{\prime}}}{{\eta \omega_{1} }}\frac{{\partial p^{\prime}}}{{r^{\prime}\partial \theta }} \hfill \\ - \frac{b}{{h^{\prime}}}(1 - \overline{\omega })r\cos \lambda_{r} \sec^{2} \lambda_{r} \hfill \\ \end{gathered} \right]\frac{{2b^{2} + bh^{\prime}}}{{2\eta \omega_{1} }}\frac{{\partial p^{\prime}}}{{r^{\prime}\partial \theta }} \hfill \\ + \left[ {\frac{{2b^{2} + bh^{\prime}}}{{2\eta \omega_{1} }}\frac{{\partial p^{\prime}}}{{r^{\prime}\partial \theta }}} \right]^{2} \hfill \\ \end{gathered} \right\} \hfill \\ + \frac{{h^{{\prime}{3}} (2b + 1)}}{12\eta }\left( {\omega_{1} \left[ \begin{gathered} \overline{\omega }\sec^{2} \lambda_{r} - \tan^{2} \lambda_{r} \hfill \\ + 1 - \frac{2b}{{h^{\prime}}}(1 - \overline{\omega })\sec^{2} \lambda_{r} \hfill \\ \end{gathered} \right]r\cos \lambda_{r} + \frac{{2b^{2} }}{\eta }\frac{{\partial p^{\prime}}}{{r^{\prime}\partial \theta }}} \right)\frac{{\partial p^{\prime}}}{{r^{\prime}\partial \theta }} \hfill \\ \end{gathered} \right\} \hfill \\ \end{gathered}$$

Introducing the non-dimensional parameters $\overline{{r^{\prime}}} = r^{\prime}/R_{2}$, $\overline{r} = r/R_{2}$, $\overline{\rho }_{r} = \rho_{r} /R_{2}$,$\overline{p^{\prime}} = p^{\prime}/p_{s}$,$\overline{h^{\prime}} = h^{\prime}/h_{0}$, $\overline{{\omega_{1} }} = \eta R_{2}^{2} \omega_{1} /(p_{s} h_{0}^{3} )$ and $\overline{{\dot{z}}}_{nut} = \eta R_{2}^{2} \dot{z}_{nut} /(p_{s} h_{0}^{3} )$, the non-dimensional form of the reynolds equation considering boundary slip can be derived as

19 $$\begin{aligned} \frac{\partial }{{\partial \overline{{r^{\prime}}} }}\left( {\overline{{r^{\prime}}} \overline{{h^{\prime}}}^{3} \frac{{\partial \overline{{p^{\prime}}} }}{{\partial \overline{{r^{\prime}}} }}} \right) + \frac{1}{{\overline{{r^{\prime}}} }}\frac{\partial }{{\partial \theta^{\prime}}}\left( {\overline{{h^{\prime}}}^{3} \frac{{\partial \overline{{p^{\prime}}} }}{{\partial \theta^{\prime}}}} \right) = & 12\rho \cos \alpha \frac{\partial }{{\partial \overline{{r^{\prime}}} }}\left( {\frac{{\overline{{r^{\prime}}} \overline{{h^{\prime}}}^{3} }}{{120\overline{\rho }_{r} }}C_{\theta 1} } \right) \\ & \quad + 6\eta \frac{\partial }{{\partial \overline{{r^{\prime}}} }}\left( {\overline{{r^{\prime}}} \overline{{h^{\prime}}} C_{r} } \right) + 6\eta C_{\theta 2} \frac{{\partial \overline{{h^{\prime}}} }}{{\partial \theta^{\prime}}} \\ & \quad \pm 12\eta \overline{{r^{\prime}}} \left( {\frac{{P(\overline{\omega } - 1)\overline{\omega }_{1} }}{{2\pi h_{0} }} + \overline{\dot{z}}_{nut} } \right)\cos \lambda_{r} \cos \alpha_{r} \\ \end{aligned}$$

where

20 $$\begin{gathered} C_{r} = \frac{{1}}{{h_{0}^{2} }}\frac{{2b^{2} }}{\eta }\frac{{\partial \overline{{p^{\prime}}} }}{{\partial \overline{{r^{\prime}}} }} \hfill \\ + \frac{b}{{h_{0}^{2} \eta }}\left\{ \begin{gathered} \frac{{p_{s}^{2} }}{{24\left( {\eta \overline{{r^{\prime}}} R_{2} } \right)^{2} }}\left[ \begin{gathered} b^{2} (\overline{h^{\prime}}h_{0} )^{5} + 4b^{3} (\overline{h^{\prime}}h_{0} )^{4} \hfill \\ + (12b^{4} + 2b^{2} )(\overline{h^{\prime}}h_{0} )^{3} \hfill \\ - 4b^{3} (\overline{h^{\prime}}h_{0} )^{2} - (48b^{5} + 24b^{4} )\overline{h^{\prime}}h_{0} \hfill \\ \end{gathered} \right]\left( {\frac{{\partial \overline{{p^{\prime}}} }}{\partial \theta }} \right)^{2} \hfill \\ \frac{{p_{s} }}{{24\eta \omega_{1} \cos \lambda_{r} \left( {\overline{{r^{\prime}}} R_{2} } \right)^{2} }}\left\{ \begin{gathered} \left[ \begin{gathered} 4b(\overline{h^{\prime}}h_{0} )^{4} + (20b^{2} + 4b + 2)(\overline{h^{\prime}}h_{0} )^{3} \hfill \\ + 24b^{3} (\overline{h^{\prime}}h_{0} )^{2} - (48b^{3} + 24b^{2} )\overline{h^{\prime}}h_{0} \hfill \\ - 96b^{4} - 48b^{3} \hfill \\ \end{gathered} \right]\overline{\omega }\sec^{2} \lambda_{r} \hfill \\ - \left[ \begin{gathered} 4b(\overline{h^{\prime}}h_{0} )^{4} + (16b^{2} + 4b + 2)(\overline{h^{\prime}}h_{0} )^{3} \hfill \\ - (8b^{2} + 4b)(\overline{h^{\prime}}h_{0} )^{2} - (48b^{3} + 24b^{2} )\overline{h^{\prime}}h_{0} \hfill \\ \end{gathered} \right]\tan^{2} \lambda_{r} \hfill \\ - \left[ \begin{gathered} 4b^{2} (\overline{h^{\prime}}h_{0} )^{3} + (24b^{3} + 8b^{2} + 4b)(\overline{h^{\prime}}h_{0} )^{2} \hfill \\ - 96b^{4} - 48b^{3} \hfill \\ \end{gathered} \right]\sec^{2} \lambda_{r} \hfill \\ + \left[ \begin{gathered} (8b^{2} + 4b + 2)(\overline{h^{\prime}}h_{0} )^{3} + (8b^{2} + 4b)(\overline{h^{\prime}}h_{0} )^{2} \hfill \\ - (48b^{3} + 24b^{2} )(\overline{h^{\prime}}h_{0} ) \hfill \\ \end{gathered} \right] \hfill \\ \end{gathered} \right\}\frac{{\partial \overline{{p^{\prime}}} }}{\partial \theta } \hfill \\ + \frac{{\omega_{1}^{2} (\overline{r} R_{2} \cos \lambda_{r} )^{2} }}{12}\left\{ \begin{gathered} \left[ \begin{gathered} - \frac{24}{{\overline{h^{\prime}}h_{0} }}b^{3} + \left( {6\overline{h^{\prime}}h_{o} - \frac{12}{{\overline{h^{\prime}}h_{o} }}} \right)b^{2} \hfill \\ + \left[ \begin{gathered} \frac{{48b^{3} }}{{\overline{h^{\prime}}h_{0} }} - \left( {12\overline{h^{\prime}}h_{0} - \frac{24}{{\overline{h^{\prime}}h_{0} }} - 24} \right)b^{2} \hfill \\ - \left( {8(\overline{h^{\prime}}h_{0} )^{2} - 12} \right)b \hfill \\ \end{gathered} \right]\overline{\omega } \hfill \\ + \left[ \begin{gathered} 3(\overline{h^{\prime}}h_{0} )^{3} + 8b(\overline{h^{\prime}}h_{0} )^{2} + (6b^{2} - 8b - 4)\overline{h^{\prime}}h_{0} \hfill \\ - \frac{24}{{\overline{h^{\prime}}h_{0} }}b^{3} - \left( {\frac{12}{{\overline{h^{\prime}}h_{0} }} - 24} \right)b^{2} - 12b \hfill \\ \end{gathered} \right]\overline{\omega }^{2} \hfill \\ \end{gathered} \right]\sec^{4} \lambda_{r} \hfill \\ + \left[ \begin{gathered} \left( \begin{gathered} 6b(\overline{h^{\prime}}h_{0} )^{2} - 32b^{2} - 16b \hfill \\ - \left( \begin{gathered} 6(\overline{h^{\prime}}h_{0} )^{3} + 8b(\overline{h^{\prime}}h_{0} )^{2} \hfill \\ - (16b + 8)\overline{h^{\prime}}h_{0} - 24b^{2} - 12b \hfill \\ \end{gathered} \right)\tan^{2} \lambda_{r} \hfill \\ \end{gathered} \right)\overline{\omega } \hfill \\ + \left( {8b(\overline{h^{\prime}}h_{0} )^{2} - 24b^{2} - 12b} \right)\tan^{2} \lambda_{r} \hfill \\ - 4b(\overline{h^{\prime}}h_{0} )^{2} + 24b^{2} + 12b \hfill \\ \end{gathered} \right]\sec^{2} \lambda_{r} \hfill \\ + \left[ \begin{gathered} \left( {3(\overline{h^{\prime}}h_{0} )^{3} - (8b + 4)\overline{h^{\prime}}h_{0} } \right)\tan^{4} \lambda_{r} \hfill \\ - 2(\overline{h^{\prime}}h_{0} )^{3} + (8b + 4)\overline{h^{\prime}}h_{0} \hfill \\ \end{gathered} \right]\tan^{2} \lambda_{r} \hfill \\ + (\overline{h^{\prime}}h_{0} )^{3} - (8b + 4)\overline{h^{\prime}}h_{0} \hfill \\ \end{gathered} \right\} \hfill \\ \end{gathered} \right\} \hfill \\ \end{gathered}$$

21 $$\begin{gathered} C_{\theta 1} = \frac{{p_{s} \left( {20b^{4} + b^{2} \overline{h^{\prime}}^{2} h_{0}^{2} } \right)}}{{2\eta^{2} \omega_{1}^{2} \cos^{2} \lambda_{r} (\overline{r}R_{2} )^{3} }}\left( {\frac{{\partial \overline{{p^{\prime}}} }}{\partial \theta }} \right)^{2} \hfill \\ { + }\left\{ \begin{gathered} \frac{{p_{s} }}{{\eta \omega_{1} \cos \lambda_{r} (\overline{r}R_{2} )^{2} }}\left[ \begin{gathered} (\overline{\omega }\sec^{2} \lambda_{r} - \tan^{2} \lambda_{r} )(10b^{2} + b\overline{h^{\prime}}h_{0} ) \hfill \\ - {{b(1 - \overline{\omega })\sec^{2} \lambda_{r} (20b^{2} + 6b\overline{h^{\prime}}h_{0} )} \mathord{\left/ {\vphantom {{b(1 - \overline{\omega })\sec^{2} \lambda_{r} (20b^{2} + 6b\overline{h^{\prime}}h_{0} )} {\left( {\overline{h^{\prime}}h_{0} } \right)}}} \right. \kern-\nulldelimiterspace} {\left( {\overline{h^{\prime}}h_{0} } \right)}} \hfill \\ + (10b^{2} - b\overline{h^{\prime}}h_{0} ) \hfill \\ \end{gathered} \right]\frac{{\partial \overline{{p^{\prime}}} }}{\partial \theta } \hfill \\ - \frac{10b}{{\overline{h^{\prime}}h_{0} }}\left[ \begin{gathered} 1 - {{b(1 - \overline{\omega })\sec^{2} \lambda_{r} } \mathord{\left/ {\vphantom {{b(1 - \overline{\omega })\sec^{2} \lambda_{r} } {\left( {\overline{h^{\prime}}h_{0} } \right)}}} \right. \kern-\nulldelimiterspace} {\left( {\overline{h^{\prime}}h_{0} } \right)}} \hfill \\ + (\overline{\omega }\sec^{2} \lambda_{r} - \tan^{2} \lambda_{r} ) \hfill \\ \end{gathered} \right](1 - \overline{\omega })\sec^{2} \lambda_{r} \hfill \\ + 3(\overline{\omega }\sec^{2} \lambda_{r} - \tan^{2} \lambda_{r} )^{2} \hfill \\ + 4(\overline{\omega }\sec^{2} \lambda_{r} - \tan^{2} \lambda_{r} ) + 3 \hfill \\ \end{gathered} \right\} \hfill \\ \end{gathered}$$

22 $$C_{\theta 2} = \left[ {\overline{\omega }\sec^{2} \lambda_{r} - \tan^{2} \lambda_{r} + 1 - {{2b(1 - \overline{\omega })\sec^{2} \lambda_{r} } \mathord{\left/ {\vphantom {{2b(1 - \overline{\omega })\sec^{2} \lambda_{r} } {\left( {\overline{h^{\prime}}h_{0} } \right)}}} \right. \kern-\nulldelimiterspace} {\left( {\overline{h^{\prime}}h_{0} } \right)}}} \right] + \frac{{2b^{2} p_{s} }}{{\eta \omega_{{1}} R_{2}^{2} \overline{r} \overline{{r^{\prime}}} \cos \lambda_{r} }}\frac{{\partial \overline{{p^{\prime}}} }}{\partial \theta }$$

## Oil film static and dynamic characteristics control equations with boundary slip

### Reynolds equation in concrete form

Introducing the axial displacement disturbances $\Delta z$ and axial velocity disturbances $\Delta \dot{z}$ into Eq. (1), the dimensionless normal film thicknesses on the left and right sides of the nut thread can be expressed by Eq. (23), where $\overline{h^{\prime}}_{L/R} = h^{\prime}_{L/R} \cos \lambda_{r} \cos \alpha_{r} /h_{0}$, $\Delta \overline{z} = \Delta z/h_{0}$ and $\Delta \overline{\dot{z}} = \Delta \dot{z}/h_{0}$:

23 $$\overline{{h^{\prime}}} = \left\{ \begin{gathered} \overline{h^{\prime}}_{L} - \Delta \overline{z} \cos \lambda_{r} \cos \alpha_{r} \hfill \\ \overline{h^{\prime}}_{R} + \Delta \overline{z} \cos \lambda_{r} \cos \alpha_{r} \hfill \\ \end{gathered} \right.$$

Neglecting the second and higher order terms, the oil film pressure of the nut thread can be expressed as:

24 $$\overline{{p^{^{\prime}} }} = \overline{{p_{0} }} { + }\Delta \overline{p} = \left\{ \begin{gathered} \overline{p}_{Ls} + \overline{p}_{Lz} \Delta \overline{z} + \overline{p}_{{L\dot{z}}} \Delta \overline{{\dot{z}}} \hfill \\ \overline{p}_{Rs} + \overline{p}_{Rz} \Delta \overline{z} + \overline{p}_{{R\dot{z}}} \Delta \overline{{\dot{z}}} \hfill \\ \end{gathered} \right.$$

Substituting Eq. (23) and Eq. (24) into Eq. (19) and separating variables, the static and perturbed reynolds can be expressed by:

25 $$\left\{ \begin{gathered} \frac{\partial }{{\partial \overline{{r^{\prime}}} }}\left( {\overline{{r^{\prime}}} \overline{{h^{\prime}}}_{L/R}^{3} \frac{{\partial \overline{p}_{Ls/Rs} }}{{\partial \overline{{r^{\prime}}} }}} \right) + \frac{1}{{\overline{{r^{\prime}}} }}\frac{\partial }{{\partial \theta^{\prime}}}\left( {\overline{{h^{\prime}}}_{L/R}^{3} \frac{{\partial \overline{p}_{Ls/Rs} }}{{\partial \theta^{\prime}}}} \right) \hfill \\ = 12M\frac{\partial }{{\partial \overline{{r^{\prime}}} }}\left[ {{{\overline{{r^{\prime}}} \overline{r}^{2} \overline{{h^{\prime}}}_{L/R}^{3} \cos^{2} \lambda_{r} C_{p\theta 1} } \mathord{\left/ {\vphantom {{\overline{{r^{\prime}}} \overline{r}^{2} \overline{{h^{\prime}}}_{L/R}^{3} \cos^{2} \lambda_{r} C_{p\theta 1} } {\left( {120\overline{\rho }_{r} } \right)}}} \right. \kern-\nulldelimiterspace} {\left( {120\overline{\rho }_{r} } \right)}}} \right] + 6M\frac{\partial }{{\partial \overline{{r^{\prime}}} }}\left[ {{{\left( {\overline{{r^{\prime}}} \overline{r}^{2} \overline{{h^{\prime}}}_{L/R} \cos^{2} \lambda_{r} C_{pr} } \right)} \mathord{\left/ {\vphantom {{\left( {\overline{{r^{\prime}}} \overline{r}^{2} \overline{{h^{\prime}}}_{L/R} \cos^{2} \lambda_{r} C_{pr} } \right)} {\overline{\rho }_{r} }}} \right. \kern-\nulldelimiterspace} {\overline{\rho }_{r} }}} \right] \hfill \\ \, + 6C_{p\theta 2} \overline{r} \overline{\omega }_{1} \cos \lambda_{r} \frac{{\partial \overline{{h^{\prime}}}_{L/R} }}{{\partial \theta^{\prime}}} \hfill \\ \frac{\partial }{{\partial \overline{{r^{\prime}}} }}\left( {\overline{{r^{\prime}}} \overline{{h^{\prime}}}_{L/R}^{3} \frac{{\partial \overline{p}_{Lz/Rz} }}{{\partial \overline{{r^{\prime}}} }} \pm 3\cos \lambda_{r} \cos \alpha_{r} \overline{{r^{\prime}}} \overline{{h^{\prime}}}_{L/R}^{2} \frac{{\partial \overline{p}_{Ls/Rs} }}{{\partial \overline{{r^{\prime}}} }}} \right) \hfill \\ + \frac{1}{{\overline{{r^{\prime}}} }}\frac{\partial }{{\partial \theta^{\prime}}}\left( {\overline{{h^{\prime}}}_{L/R}^{3} \frac{{\partial \overline{p}_{Lz/Rz} }}{{\partial \theta^{\prime}}} \pm 3\cos \lambda_{r} \cos \alpha_{r} \overline{{h^{\prime}}}_{L/R}^{2} \frac{{\partial \overline{p}_{Ls/Rs} }}{{\partial \theta^{\prime}}}} \right) \hfill \\ = 36M\frac{\partial }{{\partial \overline{{r^{\prime}}} }}\left[ {{{\overline{{r^{\prime}}} \overline{r}^{2} \overline{{h^{\prime}}}_{L/R}^{2} \cos \alpha_{r} \cos^{3} \lambda_{r} C_{p\theta 1} } \mathord{\left/ {\vphantom {{\overline{{r^{\prime}}} \overline{r}^{2} \overline{{h^{\prime}}}_{L/R}^{2} \cos \alpha_{r} \cos^{3} \lambda_{r} C_{p\theta 1} } {\left( {120\overline{\rho }_{r} } \right)}}} \right. \kern-\nulldelimiterspace} {\left( {120\overline{\rho }_{r} } \right)}}} \right]{ + }6M\frac{\partial }{{\partial \overline{{r^{\prime}}} }}\left( {{{\overline{{r^{\prime}}} \overline{r}^{2} \cos^{3} \lambda_{r} \cos \alpha_{r} C_{pr} } \mathord{\left/ {\vphantom {{\overline{{r^{\prime}}} \overline{r}^{2} \cos^{3} \lambda_{r} \cos \alpha_{r} C_{pr} } {\overline{\rho }_{r} }}} \right. \kern-\nulldelimiterspace} {\overline{\rho }_{r} }}} \right) \hfill \\ \frac{\partial }{{\partial \overline{{r^{\prime}}} }}\left( {\overline{{r^{\prime}}} \overline{{h^{\prime}}}_{L/R}^{3} \frac{{\partial \overline{p}_{{L\dot{z}/R\dot{z}}} }}{{\partial \overline{{r^{\prime}}} }}} \right) + \frac{1}{{\overline{{r^{\prime}}} }}\frac{\partial }{{\partial \theta^{\prime}}}\left( {\overline{{h^{\prime}}}_{L/R}^{3} \frac{{\partial \overline{p}_{{L\dot{z}/R\dot{z}}} }}{{\partial \theta^{\prime}}}} \right) = \pm 12\eta \overline{{r^{\prime}}} \cos \lambda \cos \alpha \hfill \\ \end{gathered} \right.$$

where

26 $$M = {{R_{2}^{2} \rho \cos \alpha \omega_{1}^{2} } \mathord{\left/ {\vphantom {{R_{2}^{2} \rho \cos \alpha \omega_{1}^{2} } {p_{s} }}} \right. \kern-\nulldelimiterspace} {p_{s} }}$$

27 $$C_{p\theta 1} = \left\{ \begin{gathered} 3(\overline{\omega }\sec^{2} \lambda_{r} - \tan^{2} \lambda_{r} )^{2} + 4(\overline{\omega }\sec^{2} \lambda_{r} - \tan^{2} \lambda_{r} ) + 3 \hfill \\ - \left[ {{{10b(1 - \overline{\omega })\sec^{2} \lambda_{r} } \mathord{\left/ {\vphantom {{10b(1 - \overline{\omega })\sec^{2} \lambda_{r} } {\left( {\overline{h^{\prime}}h_{0} } \right)}}} \right. \kern-\nulldelimiterspace} {\left( {\overline{h^{\prime}}h_{0} } \right)}}} \right]\left[ \begin{gathered} 1 - {{b(1 - \overline{\omega })\sec^{2} \lambda_{r} } \mathord{\left/ {\vphantom {{b(1 - \overline{\omega })\sec^{2} \lambda_{r} } {\left( {\overline{h^{\prime}}h_{0} } \right)}}} \right. \kern-\nulldelimiterspace} {\left( {\overline{h^{\prime}}h_{0} } \right)}} \hfill \\ + (\overline{\omega }\sec^{2} \lambda_{r} - \tan^{2} \lambda_{r} ) \hfill \\ \end{gathered} \right] \hfill \\ \end{gathered} \right\}$$

28 $$C_{p\theta 2} = \left[ {\overline{\omega }\sec^{2} \lambda_{r} - \tan^{2} \lambda_{r} - {{2b(1 - \overline{\omega })\sec^{2} \lambda_{r} } \mathord{\left/ {\vphantom {{2b(1 - \overline{\omega })\sec^{2} \lambda_{r} } {\left( {\overline{h^{\prime}}h_{0} } \right) + 1}}} \right. \kern-\nulldelimiterspace} {\left( {\overline{h^{\prime}}h_{0} } \right) + 1}}} \right]$$

29 $$C_{pr} = \frac{b}{{12h_{0}^{2} }}\left\{ \begin{gathered} \left[ \begin{gathered} 6b^{2} \overline{h^{\prime}}h_{0} - \frac{{24b^{3} }}{{\overline{h^{\prime}}h_{0} }} - \frac{{12b^{2} }}{{\overline{h^{\prime}}h_{0} }} - \left( \begin{gathered} 12b^{2} \overline{h^{\prime}}h_{0} + 8b(\overline{h^{\prime}}h_{0} )^{2} - 24b^{2} \hfill \\ - 12b - \frac{48}{{\overline{h^{\prime}}h_{0} }}b^{3} - \frac{{24b^{2} }}{{\overline{h^{\prime}}h_{0} }} \hfill \\ \end{gathered} \right)\overline{\omega } \hfill \\ + \left( \begin{gathered} 6b^{2} \overline{h^{\prime}}h_{0} - \frac{{24b^{3} }}{{\overline{h^{\prime}}h_{0} }} - \frac{{12b^{2} }}{{\overline{h^{\prime}}h_{0} }} + 3(\overline{h^{\prime}}h_{0} )^{3} \hfill \\ - 8b\overline{h^{\prime}}h_{0} - 4\overline{h^{\prime}}h_{0} + 8b(\overline{h^{\prime}}h_{0} )^{2} - 24b^{2} - 12b \hfill \\ \end{gathered} \right)\overline{\omega }^{2} \hfill \\ \end{gathered} \right]\sec^{4} \lambda_{r} \hfill \\ + \left[ \begin{gathered} \left( \begin{gathered} 6b(\overline{h^{\prime}}h_{0} )^{2} - 32b^{2} - 16b \hfill \\ - (8b(\overline{h^{\prime}}h_{0} )^{2} - 24b^{2} - 12b \hfill \\ + 6(\overline{h^{\prime}}h_{0} )^{3} - 16b\overline{h^{\prime}}h_{0} - 8\overline{h^{\prime}}h_{0} )\tan^{2} \lambda_{r} \hfill \\ \end{gathered} \right)\overline{\omega } \hfill \\ - (4b(\overline{h^{\prime}}h_{0} )^{2} - 24b^{2} - 12b) \hfill \\ + (8b(\overline{h^{\prime}}h_{0} )^{2} - 24b^{2} - 12b)\tan^{2} \lambda_{r} \hfill \\ \end{gathered} \right]\sec^{2} \lambda \hfill \\ + \left[ \begin{gathered} (3(\overline{h^{\prime}}h_{0} )^{3} - 8b\overline{h^{\prime}}h_{0} - 4\overline{h^{\prime}}h_{0} )\tan^{4} \lambda_{r} \hfill \\ - (2(\overline{h^{\prime}}h_{0} )^{3} - 8b\overline{h^{\prime}}h_{0} - 4\overline{h^{\prime}}h_{0} ) \hfill \\ \end{gathered} \right]\tan^{2} \lambda_{r} \hfill \\ + ((\overline{h^{\prime}}h_{0} )^{3} - 8b\overline{h^{\prime}}h_{0} - 4\overline{h^{\prime}}h_{0} ) \hfill \\ \end{gathered} \right\}$$

### Flow continuity equation

The dimensionless form of flow through the capillary restrictor is derived as

30 $$\overline{Q}_{c} = {{\overline{C}_{res} \left( {1 - \overline{P}_{res} } \right)} \mathord{\left/ {\vphantom {{\overline{C}_{res} \left( {1 - \overline{P}_{res} } \right)} {12}}} \right. \kern-\nulldelimiterspace} {12}}$$

where $$\overline{C}_{res} = {{3\pi d_{c}^{4} } \mathord{\left/ {\vphantom {{3\pi d_{c}^{4} } {\left( {32h_{0}^{3} L_{c} } \right)}}} \right. \kern-\nulldelimiterspace} {\left( {32h_{0}^{3} L_{c} } \right)}}$$.

Neglecting the second and higher terms, the perturbed groove pressure can be given by

31 $$\overline{P}_{res} = \overline{P}_{ls/rs} + \overline{P}_{lz/rz} \Delta \overline{z} + \overline{P}_{{l\dot{z}/r\dot{z}}} \Delta \overline{{\dot{z}}}$$

**Figure 8:**
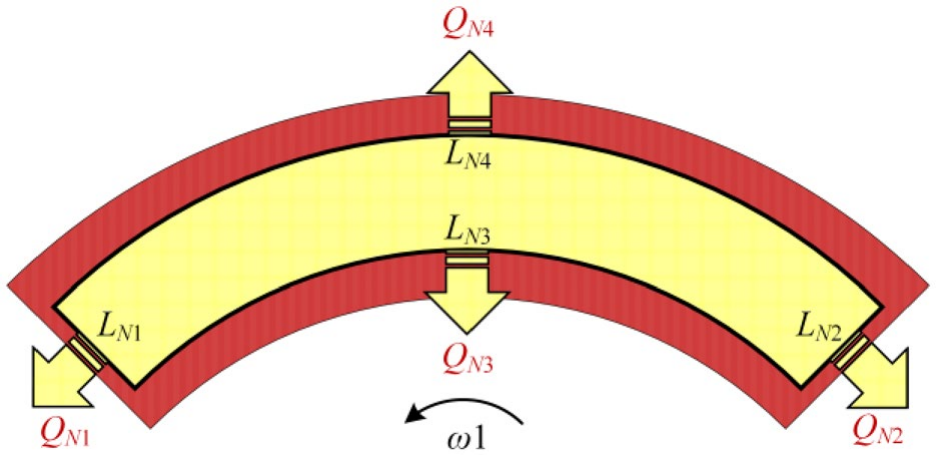
Outflow diagram of the recess.

Figure 8 shows the outflow of the *N*th spiral oil groove of the *M*th thread, which can be calculated by

32 $$\begin{aligned} \overline{Q}_{MN} = & \int_{{L_{N1} }} {\left[ {\frac{{\overline{{h^{\prime}}}^{3} }}{{12\overline{{r^{\prime}}} }}\frac{{\partial \overline{{p^{\prime}}} }}{{\partial \theta^{\prime}}}\left| \begin{gathered} \hfill \\_{{L_{N1} }} \hfill \\ \end{gathered} \right. - \frac{{\overline{r} \overline{{\omega_{1} }} \overline{{h^{\prime}}} \cos \lambda_{r} C_{p\theta 2} }}{2}} \right]} d\overline{{r^{\prime}}} \\ & \quad + \int_{{L_{N2} }} {\left[ { - \frac{{\overline{{h^{\prime}}}^{3} }}{{12\overline{{r^{\prime}}} }}\frac{{\partial \overline{{p^{\prime}}} }}{{\partial \theta^{\prime}}}\left| \begin{gathered} \hfill \\_{{L_{N2} }} \hfill \\ \end{gathered} \right. + \frac{{\overline{r} \overline{{\omega_{1} }} \overline{{h^{\prime}}} \cos \lambda_{r} C_{p\theta 2} }}{2}} \right]} d\overline{{r^{\prime}}} \\ & \quad + \int_{{L_{N3} }} {\left[ {\frac{{\overline{{h^{\prime}}}^{3} \overline{{r^{\prime}}} }}{12}\frac{{\partial \overline{{p^{\prime}}} }}{{\partial \overline{{r^{\prime}}} }}\left| \begin{gathered} \hfill \\_{{L_{N3} }} \hfill \\ \end{gathered} \right. - \frac{{M\overline{{r^{\prime}}} \overline{r}^{2} \overline{{h^{\prime}}}^{3} \cos^{2} \lambda_{r} C_{p\theta 1} }}{{120\overline{\rho }_{r} }} - \frac{{M\overline{{r^{\prime}}} \overline{r}^{2} \overline{{h^{\prime}_{s} }} \cos^{2} \lambda_{r} C_{pr} }}{{2\overline{\rho }_{r} }}} \right]} d\theta^{\prime} \\ & \quad + \int_{{L_{N4} }} {\left[ { - \frac{{\overline{{h^{\prime}}}^{3} \overline{{r^{\prime}}} }}{12}\frac{{\partial \overline{{p^{\prime}}} }}{{\partial \overline{{r^{\prime}}} }}\left| \begin{gathered} \hfill \\_{{L_{N4} }} \hfill \\ \end{gathered} \right. + \frac{{M\overline{{r^{\prime}}} \overline{r}^{2} \overline{{h^{\prime}}}^{3} \cos^{2} \lambda_{r} C_{p\theta 1} }}{{120\overline{\rho }_{r} }} + \frac{{M\overline{{r^{\prime}}} \overline{r}^{2} \overline{{h^{\prime}_{s} }} \cos^{2} \lambda_{r} C_{pr} }}{{2\overline{\rho }_{r} }}} \right]} d\theta^{\prime} \\ \end{aligned}$$

According to the flow conservation equation, the outflow of the capillary restrictor is the same as that of the spiral groove:

33 $$\overline{Q}_{c} = \overline{Q}_{MN}$$

Substituting Eqs. (23), (31) into Eq. (32), the static and perturbed flow conservation equation can be calculated by

34 $$\begin{aligned} \overline{Q}_{MNs} = & \int_{{L_{N1} }} {\left[ {\frac{{\overline{{h^{\prime}}}_{L/R}^{3} }}{{12\overline{{r^{\prime}}} }}\frac{{\partial \overline{p}_{Ls/Rs} }}{{\partial \theta^{\prime}}}\left| \begin{gathered} \hfill \\_{{L_{N1} }} \hfill \\ \end{gathered} \right. - \frac{{\overline{r} \overline{\omega }_{1} \overline{{h^{\prime}}}_{L/R} \cos \lambda_{r} C_{p\theta 2} }}{2}} \right]} d\overline{{r^{\prime}}} \\ & \quad + \int_{{L_{N2} }} {\left[ { - \frac{{\overline{{h^{\prime}}}_{L/R}^{3} }}{{12\overline{{r^{\prime}}} }}\frac{{\partial \overline{p}_{Ls/Rs} }}{{\partial \theta^{\prime}}}\left| \begin{gathered} \hfill \\_{{L_{N2} }} \hfill \\ \end{gathered} \right. + \frac{{\overline{r} \overline{\omega }_{1} \overline{{h^{\prime}}}_{L/R} \cos \lambda_{r} C_{p\theta 2} }}{2}} \right]} d\overline{{r^{\prime}}} \\ & \quad + \int_{{L_{N3} }} {\left[ {\frac{{\overline{{h^{\prime}}}_{L/R}^{3} \overline{{r^{\prime}}} }}{12}\frac{{\partial \overline{p}_{Ls/Rs} }}{{\partial \overline{{r^{\prime}}} }}\left| \begin{gathered} \hfill \\_{{L_{N3} }} \hfill \\ \end{gathered} \right. - \frac{{M\overline{{r^{\prime}}} \overline{r}^{2} \overline{{h^{\prime}}}_{L/R}^{3} \cos^{2} \lambda_{r} C_{p\theta 1} }}{{120\overline{\rho }_{r} }} - \frac{{M\overline{{r^{\prime}}} \overline{r}^{2} \overline{{h^{\prime}}}_{L/R} \cos^{2} \lambda_{r} C_{pr} }}{{2\overline{\rho }_{r} }}} \right]} d\theta^{\prime} \\ & \quad + \int_{{L_{N4} }} {\left[ { - \frac{{\overline{{h^{\prime}}}_{L/R}^{3} \overline{{r^{\prime}}} }}{12}\frac{{\partial \overline{p}_{Ls/Rs} }}{{\partial \overline{{r^{\prime}}} }}\left| \begin{gathered} \hfill \\_{{L_{N4} }} \hfill \\ \end{gathered} \right. + \frac{{M\overline{{r^{\prime}}} \overline{r}^{2} \overline{{h^{\prime}}}_{L/R}^{3} \cos^{2} \lambda_{r} C_{p\theta 1} }}{{120\overline{\rho }_{r} }} + \frac{{M\overline{{r^{\prime}}} \overline{r}^{2} \overline{{h^{\prime}}}_{L/R} \cos^{2} \lambda_{r} C_{pr} }}{{2\overline{\rho }_{r} }}} \right]} d\theta^{\prime} \\ \end{aligned}$$

35 $$\begin{aligned} \overline{Q}_{MNz} = & \int_{{L_{N1} }} {\left[ \begin{gathered} \frac{{\overline{{h^{\prime}}}_{L/R}^{3} }}{{12\overline{{r^{\prime}}} }}\frac{{\partial \overline{p}_{Lz/Rz} }}{{\partial \theta^{\prime}}}\left| \begin{gathered} \hfill \\_{{L_{N1} }} \hfill \\ \end{gathered} \right. \pm \cos \lambda_{r} \cos \alpha \frac{{\overline{{h^{\prime}}}_{L/R}^{2} }}{{4\overline{{r^{\prime}}} }}\frac{{\partial \overline{p}_{Ls/Rs} }}{{\partial \theta^{\prime}}}\left| \begin{gathered} \hfill \\_{{L_{N1} }} \hfill \\ \end{gathered} \right. \hfill \\ \mp {{\overline{r} \cos^{2} \lambda_{r} \cos \alpha \overline{\omega }_{1} C_{p\theta 2} } \mathord{\left/ {\vphantom {{\overline{r} \cos^{2} \lambda_{r} \cos \alpha \overline{\omega }_{1} C_{p\theta 2} } 2}} \right. \kern-\nulldelimiterspace} 2} \hfill \\ \end{gathered} \right]} d\overline{{r^{\prime}}} \\ & \quad + \int_{{L_{N2} }} {\left[ \begin{gathered} - \frac{{\overline{{h^{\prime}}}_{L/R}^{3} }}{{12\overline{{r^{\prime}}} }}\frac{{\partial \overline{p}_{Lz/Rz} }}{{\partial \theta^{\prime}}}\left| \begin{gathered} \hfill \\_{{L_{N2} }} \hfill \\ \end{gathered} \right. \mp \cos \lambda_{r} \cos \alpha \frac{{\overline{{h^{\prime}}}_{L/R}^{2} }}{{4\overline{{r^{\prime}}} }}\frac{{\partial \overline{p}_{Ls/Rs} }}{{\partial \theta^{\prime}}}\left| \begin{gathered} \hfill \\_{{L_{N2} }} \hfill \\ \end{gathered} \right. \hfill \\ \pm {{\overline{r} \cos^{2} \lambda_{r} \cos \alpha \overline{\omega }_{1} C_{p\theta 2} } \mathord{\left/ {\vphantom {{\overline{r} \cos^{2} \lambda_{r} \cos \alpha \overline{\omega }_{1} C_{p\theta 2} } 2}} \right. \kern-\nulldelimiterspace} 2} \hfill \\ \end{gathered} \right]} d\overline{{r^{\prime}}} \\ & \quad + \int_{{L_{N3} }} {\left[ \begin{gathered} \frac{{\overline{{r^{\prime}}} \overline{{h^{\prime}}}_{L/R}^{3} }}{12}\frac{{\partial \overline{p}_{Lz/Rz} }}{{\partial \overline{{r^{\prime}}} }}\left| \begin{gathered} \hfill \\_{{L_{N3} }} \hfill \\ \end{gathered} \right. \pm \frac{{\overline{{r^{\prime}}} \cos \lambda_{r} \cos \alpha \overline{{h^{\prime}}}_{L/R}^{2} }}{4}\frac{{\partial \overline{p}_{Ls/Rs} }}{{\partial \overline{{r^{\prime}}} }}\left| \begin{gathered} \hfill \\_{{L_{N3} }} \hfill \\ \end{gathered} \right. \hfill \\ \mp \overline{{r^{\prime}}} \overline{r}^{2} \cos^{3} \lambda_{r} \cos \alpha M\left( {{{\overline{{h^{\prime}}}_{L/R}^{2} C_{p\theta 1} } \mathord{\left/ {\vphantom {{\overline{{h^{\prime}}}_{L/R}^{2} C_{p\theta 1} } {\left( {40\overline{\rho }_{r} } \right)}}} \right. \kern-\nulldelimiterspace} {\left( {40\overline{\rho }_{r} } \right)}} + {{C_{pr} } \mathord{\left/ {\vphantom {{C_{pr} } {\left( {2\overline{\rho }_{r} } \right)}}} \right. \kern-\nulldelimiterspace} {\left( {2\overline{\rho }_{r} } \right)}}} \right) \hfill \\ \end{gathered} \right]} d\theta^{\prime} \\ & \quad + \int_{{L_{N4} }} {\left[ \begin{gathered} - \frac{{\overline{{r^{\prime}}} \overline{{h^{\prime}}}_{L/R}^{3} }}{12}\frac{{\partial \overline{p}_{Lz/Rz} }}{{\partial \overline{{r^{\prime}}} }}\left| \begin{gathered} \hfill \\_{{L_{N4} }} \hfill \\ \end{gathered} \right. \mp \frac{{\overline{{r^{\prime}}} \cos \lambda_{r} \cos \alpha \overline{{h^{\prime}}}_{L/R}^{2} }}{4}\frac{{\partial \overline{p}_{Ls/Rs} }}{{\partial \overline{{r^{\prime}}} }}\left| \begin{gathered} \hfill \\_{{L_{N4} }} \hfill \\ \end{gathered} \right. \hfill \\ \pm \overline{{r^{\prime}}} \overline{r}^{2} \cos^{3} \lambda_{r} \cos \alpha M\left( {{{\overline{{h^{\prime}}}_{L/R}^{2} C_{p\theta 1} } \mathord{\left/ {\vphantom {{\overline{{h^{\prime}}}_{L/R}^{2} C_{p\theta 1} } {\left( {40\overline{\rho }_{r} } \right)}}} \right. \kern-\nulldelimiterspace} {\left( {40\overline{\rho }_{r} } \right)}} + {{C_{pr} } \mathord{\left/ {\vphantom {{C_{pr} } {\left( {2\overline{\rho }_{r} } \right)}}} \right. \kern-\nulldelimiterspace} {\left( {2\overline{\rho }_{r} } \right)}}} \right) \hfill \\ \end{gathered} \right]} d\theta^{\prime} \\ \end{aligned}$$

36 $$\begin{aligned} \overline{Q}_{{MN\dot{z}}} = & \int_{{L_{N1} }} {\left[ {\frac{{\overline{{h^{\prime}}}_{L/R}^{3} }}{{12\overline{{r^{\prime}}} }}\frac{{\partial \overline{p}_{{L\dot{z}/R\dot{z}}} }}{{\partial \theta^{\prime}}}\left| \begin{gathered} \hfill \\_{{L_{N1} }} \hfill \\ \end{gathered} \right.} \right]} d\overline{{r^{\prime}}} + \int_{{L_{N2} }} {\left[ { - \frac{{\overline{{h^{\prime}}}_{L/R}^{3} }}{{12\overline{{r^{\prime}}} }}\frac{{\partial \overline{p}_{{L\dot{z}/R\dot{z}}} }}{{\partial \theta^{\prime}}}\left| \begin{gathered} \hfill \\_{{L_{N2} }} \hfill \\ \end{gathered} \right.} \right]} d\overline{{r^{\prime}}} \\ & \quad + \int_{{L_{N3} }} {\left[ {\frac{{\overline{{h^{\prime}}}_{L/R}^{3} }}{12}\overline{{r^{\prime}}} \frac{{\partial \overline{p}_{{L\dot{z}/R\dot{z}}} }}{{\partial \overline{{r^{\prime}}} }}\left| \begin{gathered} \hfill \\_{{L_{N3} }} \hfill \\ \end{gathered} \right.} \right]} d\theta^{\prime} + \int_{{L_{N4} }} {\left[ { - \frac{{\overline{{h^{\prime}}}_{L/R}^{3} }}{12}\overline{{r^{\prime}}} \frac{{\partial \overline{p}_{{L\dot{z}/R\dot{z}}} }}{{\partial \overline{{r^{\prime}}} }}\left| \begin{gathered} \hfill \\_{{L_{N4} }} \hfill \\ \end{gathered} \right.} \right]} d\theta^{\prime} \\ \end{aligned}$$

### Axial load capacity, stiffness coefficient and damping coefficient

The dimensionless form of the oil film bearing capacity can be written as

37 $$\overline{W} = \int_{0}^{{\theta_{\psi } \times N}} {\int_{{\overline{{R^{\prime}}}_{1} }}^{{\overline{{R^{\prime}}}_{2} }} {\left( {\overline{p}_{Rs} - \overline{p}_{Ls} } \right)} } \overline{{r^{\prime}}} \cos \lambda_{r} \cos \alpha_{r} d\theta^{\prime}d\overline{{r^{\prime}}}$$

The dimensionless form of the axial stiffness of the oil film is given as

38 $$\overline{K} = \int_{0}^{{\theta_{\psi } \times N}} {\int_{{\overline{{R^{\prime}}}_{1} }}^{{\overline{{R^{\prime}}}_{2} }} {\left( {\overline{p}_{Rz} - \overline{p}_{Lz} } \right)} } \overline{{r^{\prime}}} \cos \lambda_{r} \cos \alpha_{r} d\theta^{\prime}d\overline{{r^{\prime}}}$$

The dimensionless form of the axial damping coefficients of the oil film can be expressed by

39 $$\overline{C} = \int_{0}^{{\theta_{\psi } \times N}} {\int_{{\overline{{R^{\prime}}}_{1} }}^{{\overline{{R^{\prime}}}_{2} }} {\left( {\overline{p}_{{R\dot{z}}} - \overline{p}_{{L\dot{z}}} } \right)} } \overline{{r^{\prime}}} \cos \lambda_{r} \cos \alpha_{r} d\theta^{\prime}d\overline{{r^{\prime}}}$$

## Simulation results and analysis of static and dynamic characteristics with boundary slip

Recent experimental studies have shown that the boundary slip parameters of fluid flow range from several nanometers to tens of microns^14^. When the thickness of oil film is much larger than the effective range of van der Waals effect^22^, the slip coefficient *b* is mainly affected by material properties such as the roughness of contact surface, the surface wettability and the surface tension. Nevertheless, the slip coefficient can not be obtained directly from the coupling of the above factors, but experimental measurement. According to the comparison table of slip coefficient summarized in Ref.^23^ and the method mentioned in Ref.^24^, the boundary slip coefficient of the DDHSNP can be evaluated. In this paper, the numerical simulation is used to study the influence of boundary slip on the micro-flow characteristics of hydrostatic lead screw. In order to further illustrate the influence, the slip coefficient *b* is taken as 0–7 μm on account of the characteristic dimensions of the hydrostatic lead screw^23^.

In this study, the operating and geometric parameters of the DDHSNP are all listed above in Table 1. The steady-state pressure distribution and disturbed pressure distribution of spiral oil film can be derived by solved reynolds equation and flow continuity equation with finite difference method. In Ref.^25^, the finite difference method has been introduced in details. In addition, the boundary conditions used for the simulation calculation of the hydrostatic lead screw have been reported in Ref.^11^.

**Table1:** Parameters of the DDHSNP.

Parameters	Value	Parameters	Value
*R*_1_	22.5 mm	*α*	10°
*R*_2_	33.5 mm	*p*_s_	5Mpa
*r*_1_	26.5 mm	*θ*_r_	70°
*r*_2_	29.5 mm	*ρ*	875 kg/m3
*N*	5	$\overline{C}_{res}$	12.733
*P*	20 mm	$\gamma$	0.5
*h*_0_	0.025 mm		

### Verification the solution of mathematical model

**Figure 9:**
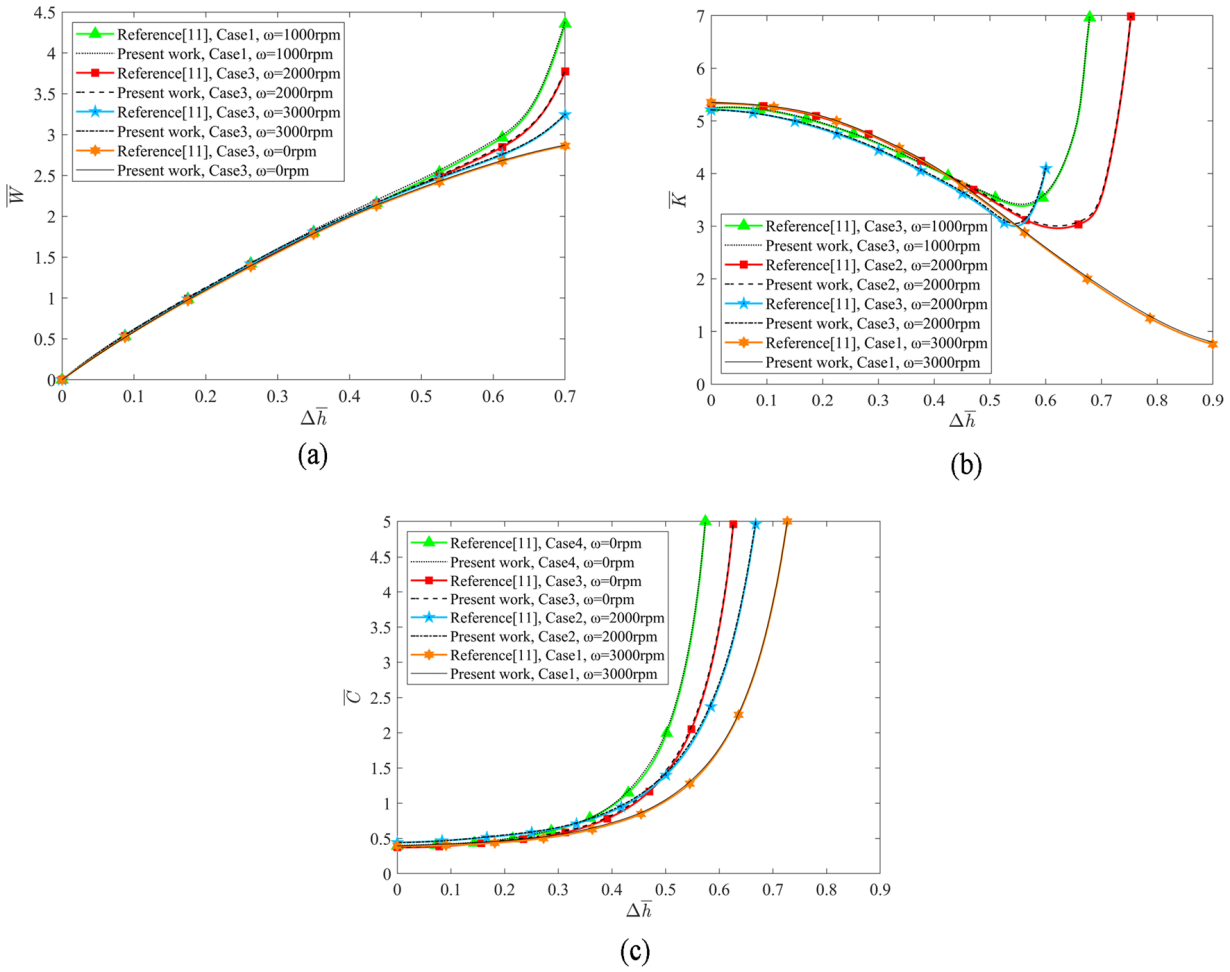
Comparison of bearing capacity, stiffness coefficient and damping coefficient of hydrostatic lead screw with Ref.^11^ (**a**) Bearing capacity (**b**) Stiffness coefficient (**c**) Damping coefficient.

When no boundary slip occurs, the solution of the mathematical model established was validated by comparing it with the results published in Ref.^11^. As shown in Fig. 9, under the premise that the parameters of the hydrostatic lead screw are the same, the calculation results of the mathematical model have a excellent consistency with that published in Ref.^11^.

### Effect of boundary slip on single driven DDHSNP

**Figure 10:**
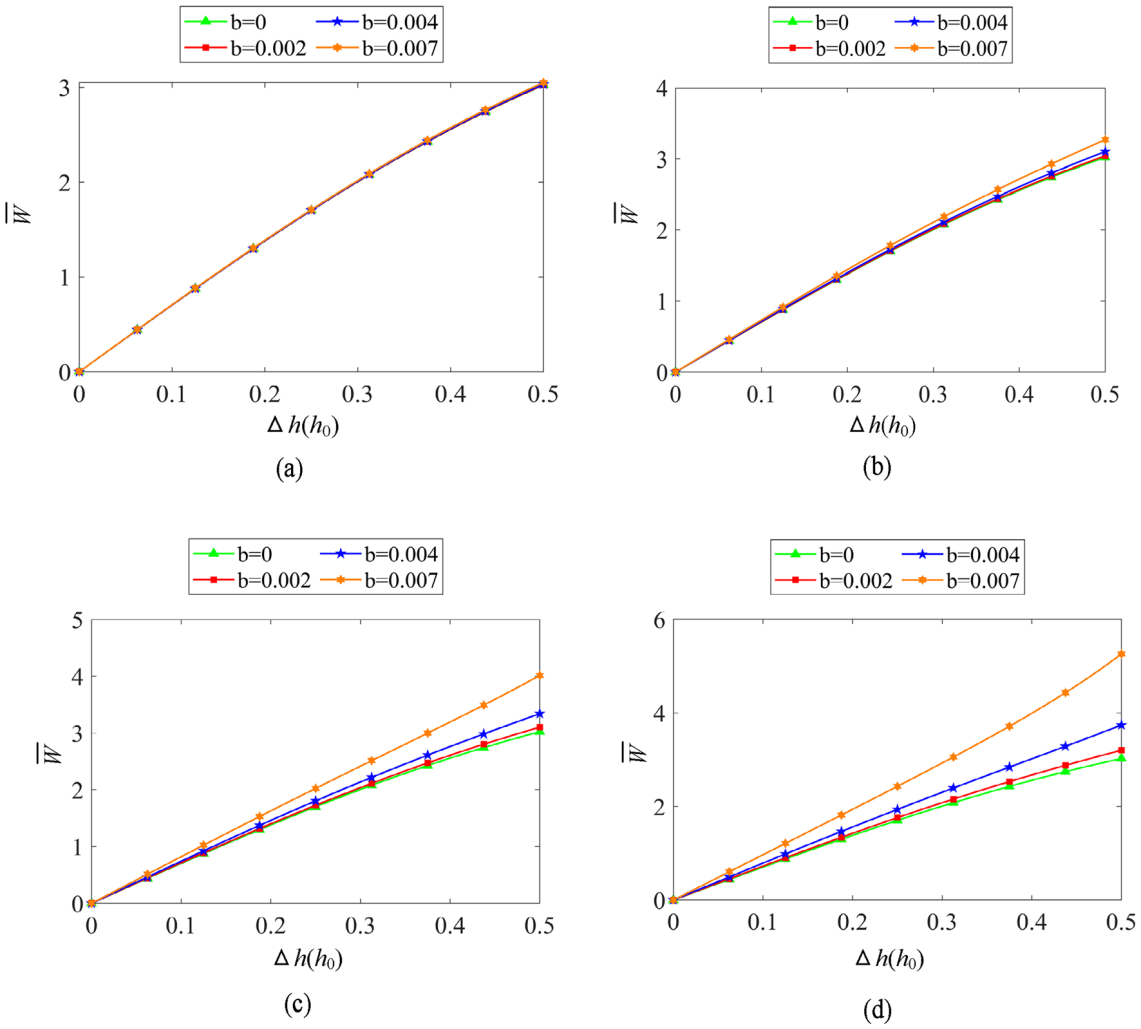
Influence of boundary slip on bearing capacity (*ω*_2_ = 0 rpm) (**a**) *ω*_1_ = 1000 rpm, (**b**) *ω*_1_ = 3000 rpm, (**c**) *ω*_1_ = 6000 rpm, (**d**) *ω*_1_ = 9000 rpm.

Figure 10a–d show the variation regularity of the axial bearing capacity for single-driven DDHSNP in considering boundary slip conditions. The slip degree is reflected by the slip coefficient *b*. When the DDHSNP operates at a constant speed, the axial bearing capacity is found to increase with increasing of the slip coefficient *b*. When the slip coefficient *b* remains constant (*b*>0), the load capacity is found to increase with increasing of the rotational speed, and the increment rate is obviously larger. As shown in Fig. 10, when Δ*h* reaches 0.5, the axial bearing capacity of the oil film with slip coefficient *b*=0.007 increases by 0.9%, 8.2%, 32.9% and 73.9% relative to the no slip condition under the rotational speeds of 1000 rpm, 3000 rpm, 6000 rpm, 9000 rpm, respectively. In summary, when the DDHSNP operates at low speed in single-drive mode (*ω*_1_ ≤ 1000 rpm), the influence of the boundary slip on the axial bearing capacity is not significant. However, when the rotational speed of *ω*_1_ reaches at a high speed range (*ω*_1_ ≥ 3000 rpm), the present of the boundary slip can improve the axial bearing capacity remarkably.

**Figure 11:**
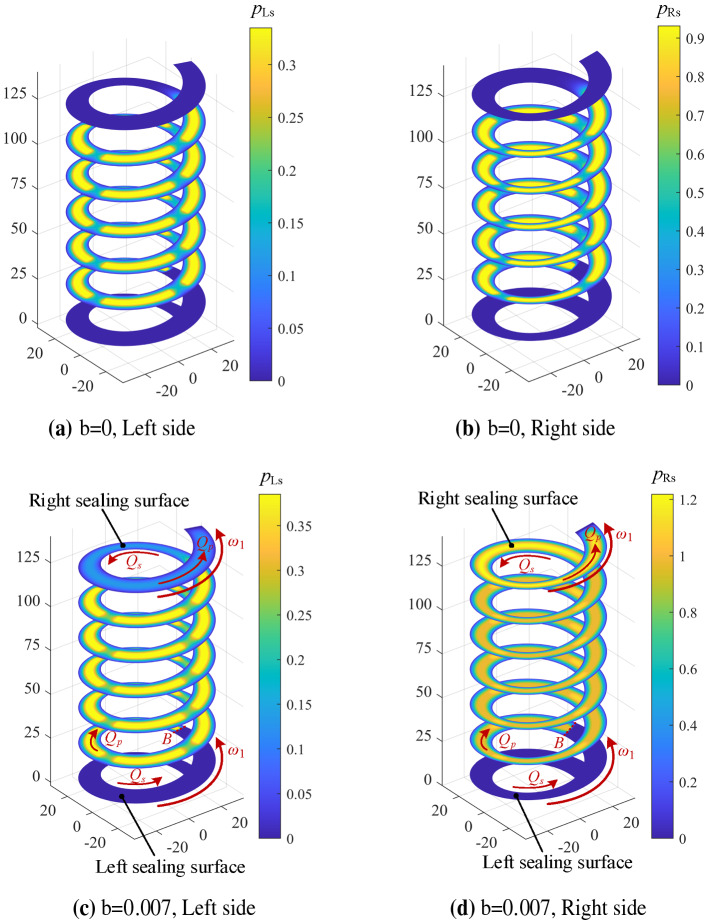
Steady-state pressure distribution under boundary slip condition with *ω*_1_ = 9000 rpm, *ω*_2_ = 0 rpm, Δ*h* = 0.5*h*_0_.

Figure 11 shows the steady-state pressure distribution of the spiral oil film with the slip coefficient *b* = 0 and the slip coefficient *b* = 0.007 under the conditions of *ω*_1_ = 9000 rpm and Δ*h* = 0.5*h*_0_. As shown in Fig. 11a,b, the axial bearing capacity mainly originates from the steady-state pressure of the spiral oil groove when no slip occurs. As seen from Fig. 11c,d, when the oil slip appears and *b* = 0.007, the steady-state pressure of the spiral oil film increases sharply, which is more obvious on the right side of sealing surface. Under the rotational speed of 9000 rpm, the flow velocity of lubricant on the right sealing surface derives from the effects of the shear flow *Q*_s_ and the pressure-induced flow *Q*_p_, so a higher flow velocity appears. For the sealing surface on the left side of the spiral oil film, *Q*_s_ and *Q*_p_ flow in opposite directions at boundary B. When *ω*_1_ = 9000 rpm, the shear flow *Q*_s_ is much greater than the pressure-induced flow *Q*_p_. It is difficult for lubricating oil to flow into the left sealing surface of the spiral oil film, the boundary slip has no effect on the left sealing surface. In addition, it can be seen from Fig. 11c,d, the pressure on the right side of the oil film is much higher than that of the left side. It can be explained that the effect of boundary slip is further obvious in small clearance due to the existence of nut displacement Δ*h*.

**Figure 12:**
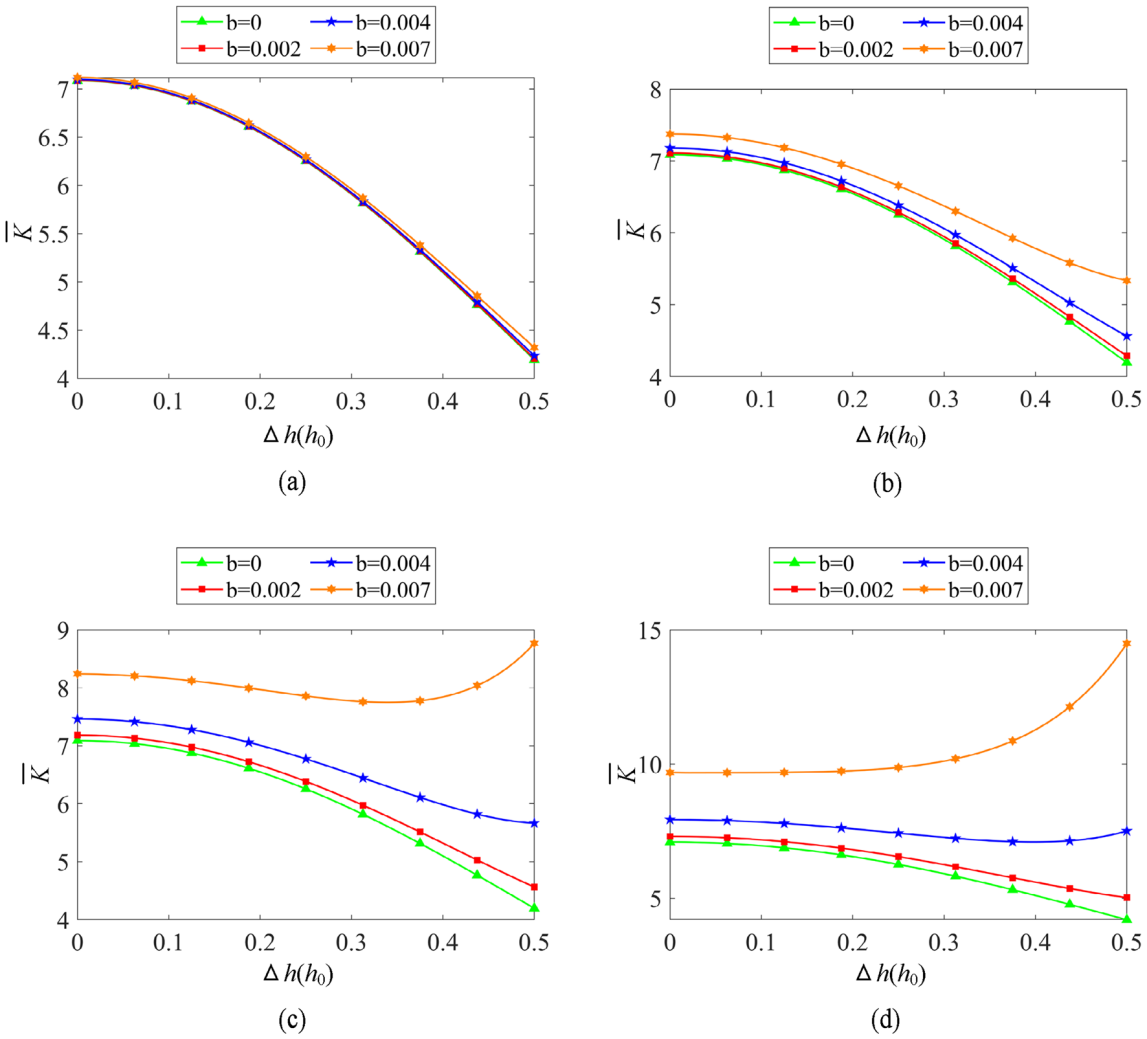
Influence of boundary slip on oil film stiffness (*ω*_2_ = 0 rpm) (**a**) *ω*_1_ = 1000 rpm, (**b**) *ω*_1_ = 3000 rpm, (**c**) *ω*_1_ = 6000 rpm, (**d**) *ω*_1_ = 9000 rpm.

As shown in Fig. 12a–d, the typical effect of boundary slip on axial stiffness is similar to that on axial bearing capacity under single-drive operation. The larger the slip coefficient *b* is, the greater the axial stiffness is. With the increase of the rotating speed, the rising tendency of axial stiffness is more distinct. As shown in Fig. 12, when Δ*h* reaches 0.5*h*_0_, the axial stiffness of the oil film with slip coefficient *b* = 0.007 increases by 3.0%, 27.2%, 108.8% and 244.9% relative to the condition without slip under the rotational speeds of 1000 rpm, 3000 rpm, 6000 rpm, 9000 rpm, respectively. It can be easily found that although the effect of boundary slip is similar on the load capacity and stiffness, the increment rate of axial stiffness is about three times larger than that of axial bearing capacity. At the same time, the existence of boundary slip changes the phenomenon that the oil film stiffness gradually decreases with the increase of the axial displacement Δ*h* under no slip condition. As depicted in Fig. 12d, the oil film stiffness even increases rapidly with the increase of the axial displacement fluctuation Δ*h* under the working conditions with large slip coefficient and high rotation speed.

**Figure 13:**
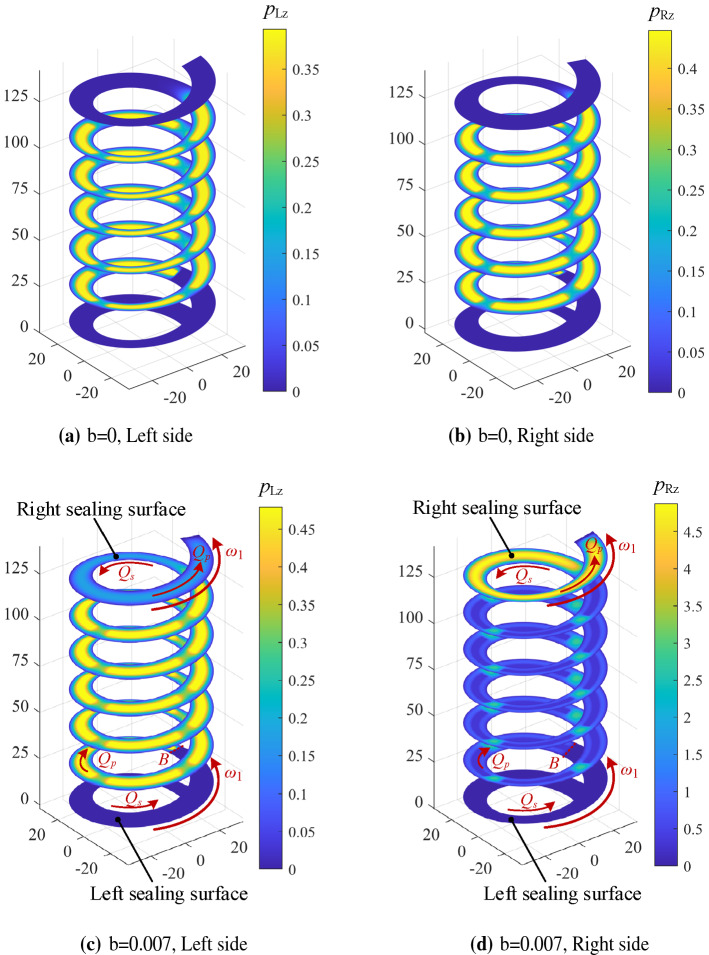
Disturbance pressure distribution under the influence of Δ*z* when *ω*_1_ = 9000 rpm, *ω*_2_ = 0 rpm, Δ*h* = 0.5 *h*_0._

As we can see in Eq. (31), the axial stiffness coefficient is derived by integrating the perturbation pressure of the oil film. The variation of the axial stiffness can be further explained by the distributions of the perturbation pressure. Figure 13 shows the distributions of spiral oil film perturbation pressure when the slip coefficient *b* = 0 and the slip coefficient *b* = 0.007 under the conditions of *ω*_1_ = 9000 rpm and Δ*h* = 0.5*h*_0_. When no oil film occurs, similar to the steady-state pressure distributions of spiral oil film, the disturbance pressure distribution is also mainly from the helical recesses (Fig. 13a,b). However, when the oil film slip occurs and the slip coefficient *b* = 0.007, the spiral oil film disturbance pressure increases significantly on the right side of screw thread (clearance decrease on this side), particularly for the right sealing surface.

**Figure 14:**
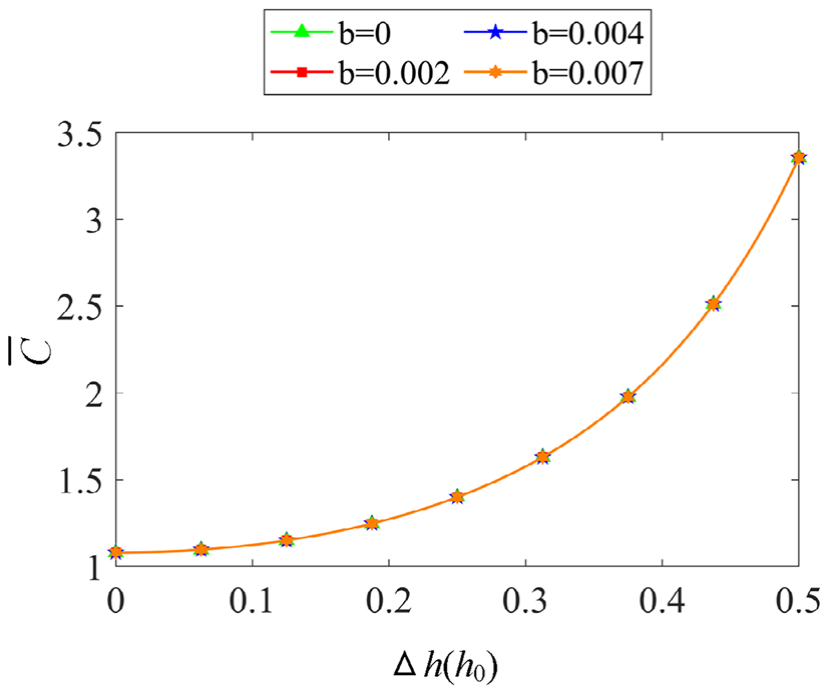
Influence of boundary slip on oil film damping coefficient (*ω*_2_ = 0 rpm).

It can be seen from Fig. 14 that the boundary slip of DDHSNP under single-drive mode hardly affects the axial damping coefficient. This is because the slip coefficient *b* is not introduced to the terms of $\overline{p}_{{L\dot{z}/R\dot{z}}}$ in Eq. (25) and Eq. (39), the existence of boundary slip has no affects on the damping coefficient during the operation of DDHSNP. Based on the above reasons, the influence of boundary slip on the damping coefficient is no longer considered in the subsequent simulation of the DDHSNP.

### Effect of boundary slip on dual-drive differential DDHSNP

Compared with the traditional hydrostatic lead screw, the advantage of DDHSNP is the dual-drive differential feed. In the dual-drive differential mode, both the lead screw motor and the nut motor can avoid the crawling zone of the motor through a high rotational speed, and micro-nano scale feed under ultra-low speed can be realized. As the power transmission medium in the DDHSNP, the axial bearing capacity and stiffness changes of spiral oil film will have an remarkable impact on the ultra-precision feed. In order to explore the influence of boundary slip on the static and dynamic characteristics of the DDHSNP, the axial bearing capacity and axial stiffness are carried out under the consideration of boundary slip and different differential conditions.

**Figure 15:**
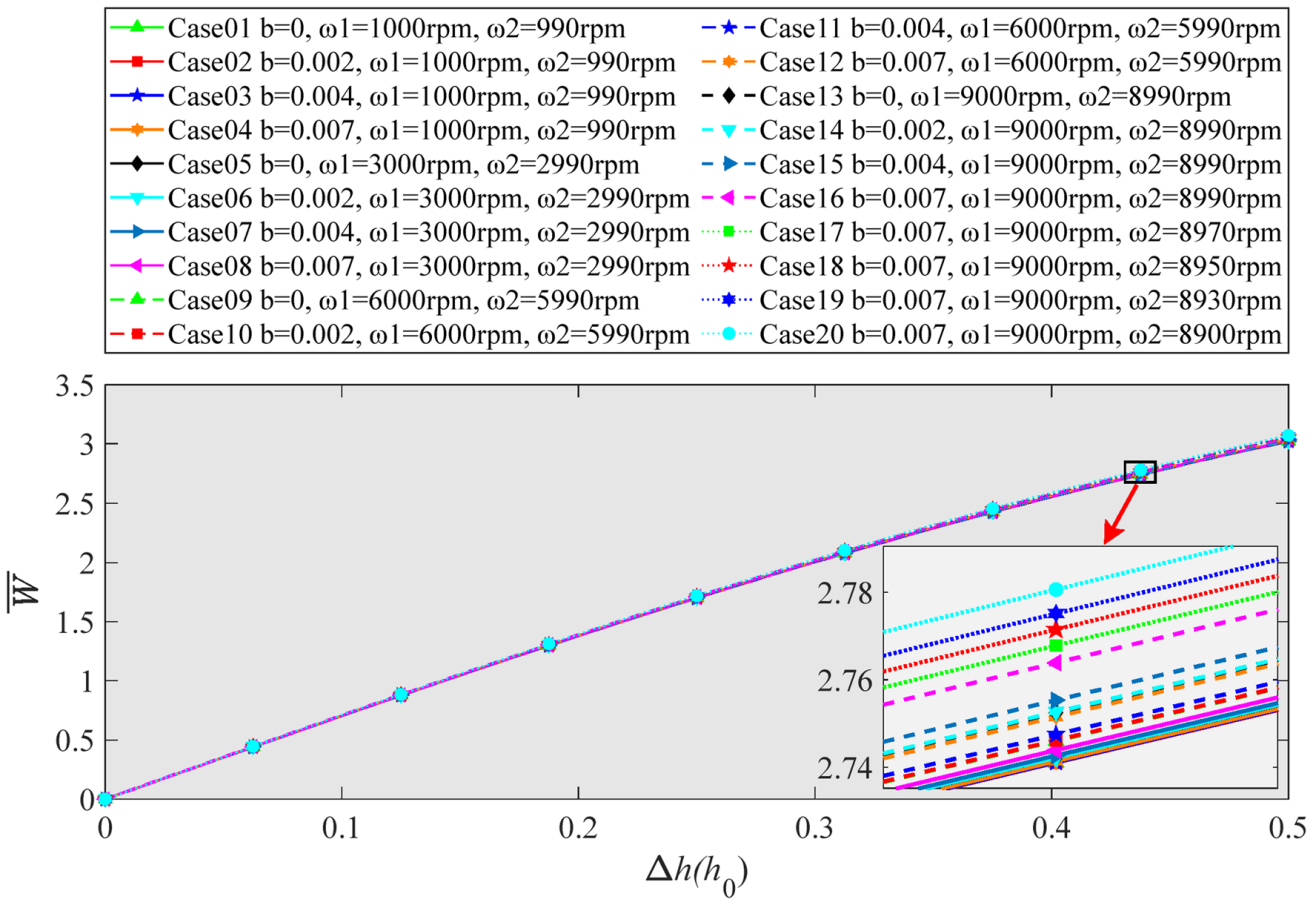
Influence of boundary slip on bearing capacity (Operation in dual drive differential mode).

Figure 15 shows the variation regularity of the axial bearing capacity with low-speed ultra-precision feed of DDHSNP in dual-drive differential operation under consideration of the boundary slip. The output differential speed of DDHSNP in Case01 ~ Case16 are all 10 rpm. The curves of Case01 ~ Case08 are almost coincident. It should be noted that when Δ*h* reaches 0.5, the dimensionless bearing capacity of Case16 increased by 0.015, 0.024, 0.027, 0.029 compared to Case12, Case08, Case04, and Case01, respectively. The maximum increase of axial bearing capacity is less than 1%. The output differential speed of DDHSNP in Case16 ~ Case20 is 10 rpm, 30 rpm, 50 rpm, 70 rpm, and 100 rpm, respectively. When Δ*h* reaches 0.5, for Case17, Case18, Case19, and Case20, the axial load capacity increased by 0.005, 0.010, 0.015, and 0.022 relative to Case16, respectively. With the increase of the output differential velocity, the maximum increase of the oil film bearing capacity of the DDHSNP is less than 1%. Most notably, compared with Case01 and Case20, even if the macro velocities *ω*_1_ and *ω*_2_ increase from *ω*_1_ = 1000 rpm and *ω*_2_ = 990 rpm to *ω*_1_ = 9000 rpm and *ω*_2_ = 8990 rpm, and the fluid flow from without boundary slip to the boundary slip coefficient *b* = 0.007, the maximum increase in the axial bearing capacity of the oil film is only 1.7%. To sum up, the existence of boundary slip has little effect on the axial bearing capacity during low-speed feed in the DDHSNP dual-drive differential mode.

**Figure 16:**
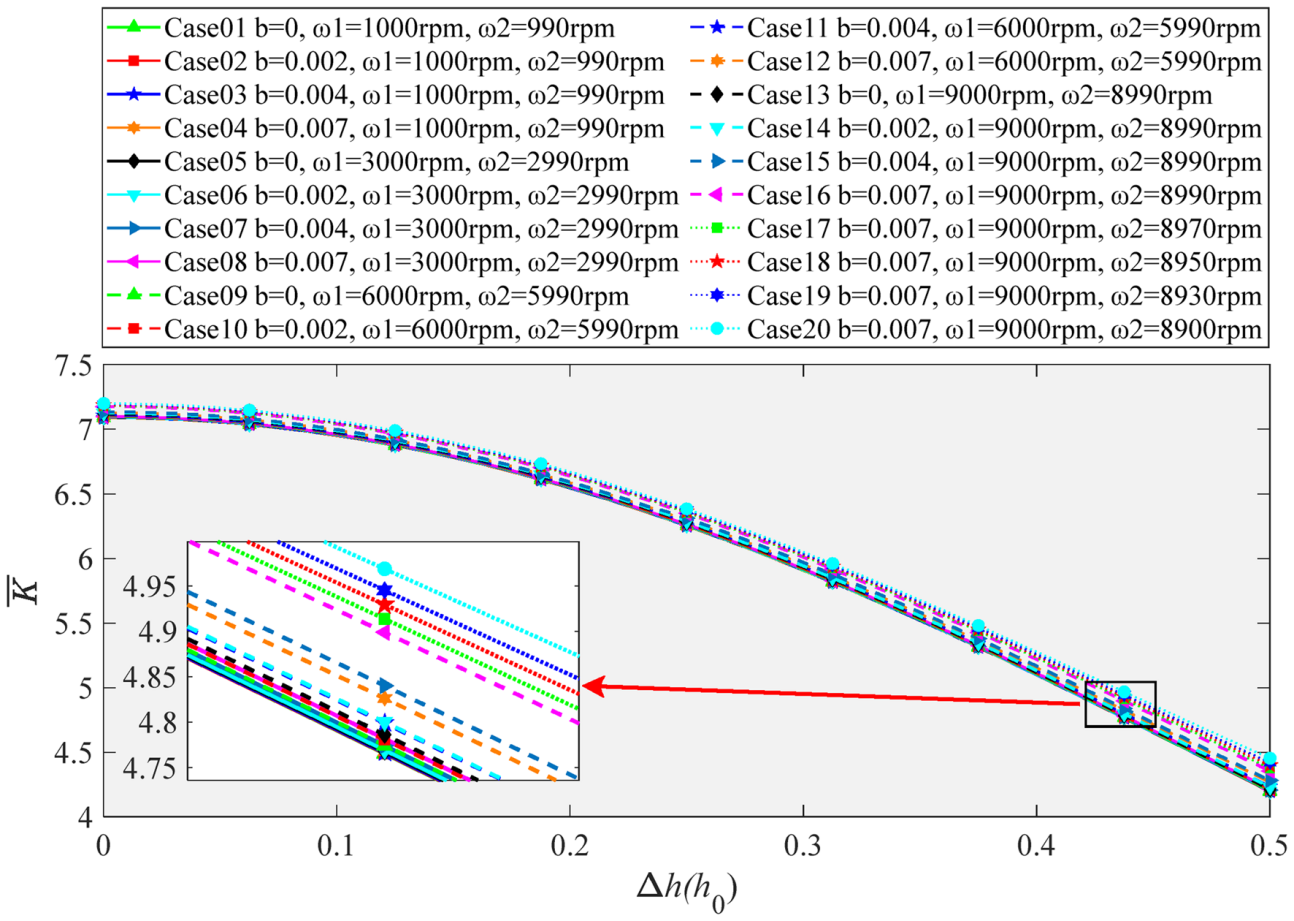
Influence of boundary slip on oil film stiffness (Operation in dual drive differential mode).

Figure 16 shows the variation regulation of oil film stiffness with low-speed ultra-precision feed of DDHSNP in dual-drive differential operation. As can see in Figs. 15 and 16, compared with the change of axial bearing capacity, the stiffness coefficient also varies slightly in Case01 ~ Case08. When Δ*h* = 0.5, the dimensionless stiffness of Case16 only enhanced by 0.087, 0.140, 0.158, 0.160 contrast to Case12, Case08, Case04, and Case01, respectively. Obviously, the maximum increase rate in axial stiffness is 3.8%. In addition, the stiffness coefficients of Case17, Case18, Case19, Case20 enhanced by 0.0212, 0.0427, 0.0647, and 0.0978, respectively, compared to Case16, with a maximum increase of 2.2%. Compared with Case01 and Case20, when the macro-motion speeds *ω*_1_ and *ω*_2_ increase greatly, the slip coefficient *b* increases from 0 to the highest value of 0.007, and the differential rotation rate increases from 10 to 100 rpm, the maximum increase of oil film stiffness is 6%. Furthermore, from the comparison between Figs. 12 and 16, it can be seen that the existence of boundary slip in the dual-drive differential mode does not change the downward tendency of the axial stiffness with the increase of Δ*h*. In summary, the existence of boundary slip can raise the axial stiffness coefficient in the dual-drive differential mode of DDHSNP. Although the increase amplitude is about three times larger than that of axial bearing capacity, the increase is still slight. It also has a trend of gradual increase with the increase of macro velocities *ω*_1_, *ω*_2_ and their differential velocities.

The effect of boundary slip on the axial bearing capacity and stiffness coefficient of the oil film in dual-drive differential mode is unobvious compared with that in single-drive mode. The reason is as follows: When the DDHSNP operates in single-drive mode, only one side of the solid–liquid interface of the oil film makes the liquid molecules to slip along the surface. When the DDHSNP runs in the dual-drive differential mode, the oil film has shear stress at the solid–liquid interface on the screw side and the nut side, the direction is the same. According to Ref.^26^, the shearing rate of solid–liquid interface decreases with the reduction of velocity difference between the lead screw and the nut, thus greatly weakening the slip degree.

## Conclusion

Aiming at the proposed novel dual-drive hydrostatic screw nut pair, the influence of boundary slip on the static and dynamic characteristics under single drive mode and double drive differential mode were studied. The main conclusions are as follows:

1. When the DDHSNP is single-driven at a low speed (the lead screw rotation rate *ω*_1_ ≤ 1000 rpm ), the boundary slip has little effect on the axial bearing capacity and stiffness. When the DDHSNP is single-driven at a high speed (the lead screw rotation rate *ω*_1_ > 1000 rpm), the axial bearing capacity of the oil film improves with the increase of the slip coefficient, and the increasing amplitude increases in multiples with the increase of the speed; The boundary slip can also improve the axial stiffness of the oil film, the increment rate of oil film axial stiffness is more obvious, which is about three times larger than that of axial bearing capacity; In addition, with the increase of rotation speed, the existence of boundary slip gradually changes the law that the oil film stiffness decreases with the increase of nut axial displacement.
2. When DDHSNP feeds in dual-drive differential mode (the rotating speed range of screw and nut is 1000 rpm ~ 9000 rpm, the range of speed difference is 10 rpm ~ 100 rpm), the existence of boundary slip can hardly improve the oil film bearing capacity, and the change rate is less than 2%; Similar to the single drive mode, the increase of stiffness coefficient under dual-drive differential mode is also about three times than that of axial bearing capacity. But the maximum increment rate of oil film stiffness is only 6%. In addition, when the boundary slip occurs, the axial bearing capacity and stiffness can gradually increases with the increase of slip coefficient, lead screw speed, nut speed and the difference speed between them.
3. No matter whether the DDHSNP is in single-drive operation or dual-drive differential operation, the existence of boundary slip has almost no effect on the oil film damping coefficient.

## List of symbols

*h*_0_: Designed axial clearance for DDHSNP (mm)
∆*h*: Displacement of nut (mm)
*h*_L_ʹ: Thickness of oil film on the nut left side (mm)
*h*_R_ʹ: Thickness of oil film on the nut right side (mm)
*θ*_*r*_: Wrap angle of the oil recess
*θ*_*s*_: Interval angle of the oil recess
*R*_1_: Inner radius of the nut (mm)
*R*_2_: Outer radius of the lead screw (mm)
*r*_1_: Inner radius of the oil recess (mm)
*r*_2_: Outer radius of the oil recess (mm)
*R*_1_ʹ: Inner radius of equivalent sector (mm)
*R*_2_ʹ: Outer radius of equivalent sector (mm)
*θ*_*ψ*_: Center angle of equivalent sector (mm)
*α*: Half angle of thread (mm)
$v_{{\theta^{{\prime }} }}$: Velocity composition in *θ*^ʹ^ direction (mm/s)
$v_{{r^{{\prime }} }}$: Velocity composition in *r*^ʹ^ direction (mm/s)
$v_{{z^{{\prime }} }}$: Velocity composition in *z*^ʹ^ direction (mm/s)
*b*: Slip coefficient
*u*_1_: Linear speed of nut rotation (mm/s)
*u*_2_: Linear speed of lead screw rotation (mm/s)
*γ*: Design pressure ratio
$\dot{z}_{nut}$: Axial velocity of nut (mm/s)
*h*ʹ: The film thickness (mm)
*λ*_*r*_: Helix angle
*η*: Viscosity of lubricating oil (Pa·s)
*ρ*: Density of lubricating oil (kg/mm^3^)
*ρ*_r_: Curvature radiusat any radius (mm)
*ω*_1_: Rotation speed of the nut (rpm)
*ω*_2_: Rotation speed of the lead screw (rpm)
*P*: Pitch (mm)
*p*ʹ: Oil film pressure (Pa)
*p*_*s*_: Oil inlet pressure (Pa)
∆*z*: Nut axial displacement disturbance
$\Delta \dot{z}$: Nut axial velocity disturbance
*N*: Number of spiral oil chambers
*M*: Number of wrapped threads
*Q*_*s*_: Flow driven by the rotation of the screw
*Q*_*p*_: Pressure driven fluid flow
*B*: Sealing surface boundary
*u*_*s*__1_: Slip velocity of nut surface in θʹ direction (mm/s)
*u*_*s*__2_: Slip velocity of lead screw surface in θʹ direction (mm/s)
*ν*_*r*__1_: Slip velocity of nut surface in θʹ direction (mm/s)
*ν*_*r*__2_: Slip velocity of lead screw surface in θʹ direction (mm/s)
*Q*_*c*_: Flow through the capillary restrictor (mm^3^/s)
*Q*_*MN*_: Outflow from helical recess (mm^3^/s)
*Q*_*MNs*_: Steady-state outflow from helical recess (mm^3^/s)
*Q*_*MNz*_: Disturbance outflow from helical recess caused by ∆z (mm^3^/s)
$Q_{{MN\dot{z}}}$: Disturbance outflow from helical recess caused by $\Delta \dot{z}$ (mm^2^)
$q_{{\theta^{{\prime }} }}$: Flow rate of unit oil film in *θ*ʹ direction (mm^3^/s)
$q_{{r^{{\prime }} }}$: Flow rate of unit oil film in *r*ʹ direction (mm^3^/s)
$q_{{z^{{\prime }} }}$: Flow rate of unit oil film in *z*ʹ direction (mm^3^/s)
*p*_*Ls*__/__*Rs*_: Oil film steady state pressure on left and right sides of nut thread (Pa)
*p*_*Lz*__/__*Rz*_: Oil film disturbance pressure on left and right sides of nut thread caused by ∆z (Pa/mm)
$p_{{L\dot{z}/R\dot{z}}}$: Oil film disturbance pressure on left and right sides of nut thread caused by $\Delta \dot{z}$ (pa/mm)
*p*_*ls*__/__*rs*_: Helical recess steady state pressure on the left and right sides of nut thread (Pa)
*p*_*lz*__/__*rz*_: Helical recess disturbance pressure on left and right sides of nut thread caused by ∆z (Pa/mm)
$p_{{l\dot{z}/r\dot{z}}}$: Helical recess disturbance pressure on left and right sides of nut thread caused by $\Delta \dot{z}$ (Pa s/mm

## Non-dimensional parameters

$$\overline{{r^{\prime}}}$$: $$r^{\prime}/R_{2}$$
$$\overline{r}$$: $$r/R_{2}$$
$$\overline{\rho }_{r}$$: $$\rho_{r} /R_{2}$$
$$\overline{p^{\prime}}$$: $$p^{\prime}/p_{s}$$
$$\overline{{\omega_{1} }}$$: $$\eta R_{2}^{2} \omega_{1} /(p_{s} h_{0}^{3} )$$
$$\overline{{\dot{z}}}_{nut}$$: $$\eta R_{2}^{2} \dot{z}_{nut} /(p_{s} h_{0}^{3} )$$
$$\overline{\omega }$$: $$\omega_{2} /\omega_{1}$$
$$M$$: $${{R_{2}^{2} \rho \cos \alpha \omega_{1}^{2} } \mathord{\left/ {\vphantom {{R_{2}^{2} \rho \cos \alpha \omega_{1}^{2} } {p_{s} }}} \right. \kern-\nulldelimiterspace} {p_{s} }}$$
$$\overline{W}$$: $${W \mathord{\left/ {\vphantom {W {p_{S} }}} \right. \kern-\nulldelimiterspace} {p_{S} }}R_{2}^{2}$$
$$\overline{K}$$: $${{Kh_{0} } \mathord{\left/ {\vphantom {{Kh_{0} } {p_{S} }}} \right. \kern-\nulldelimiterspace} {p_{S} }}R_{2}^{2}$$
$$\overline{C}$$: $${{Ch_{0}^{3} } \mathord{\left/ {\vphantom {{Ch_{0}^{3} } \eta }} \right. \kern-\nulldelimiterspace} \eta }R_{2}^{4}$$
$$\overline{Q}_{c}$$: $${{Q_{c} \eta } \mathord{\left/ {\vphantom {{Q_{c} \eta } {p_{s} h_{0}^{3} }}} \right. \kern-\nulldelimiterspace} {p_{s} h_{0}^{3} }}$$
$$\overline{p}_{Ls/Rs}$$: $${{p_{Ls/Rs} } \mathord{\left/ {\vphantom {{p_{Ls/Rs} } {P_{s} }}} \right. \kern-\nulldelimiterspace} {P_{s} }}$$
$$\overline{p}_{Lz/Rz}$$: $${{p_{Lz/Rz} h_{0} } \mathord{\left/ {\vphantom {{p_{Lz/Rz} h_{0} } {P_{s} }}} \right. \kern-\nulldelimiterspace} {P_{s} }}$$
$$\overline{p}_{{L\dot{z}/R\dot{z}}}$$: $${{p_{{L\dot{z}/R\dot{z}}} h_{0}^{3} } \mathord{\left/ {\vphantom {{p_{{L\dot{z}/R\dot{z}}} h_{0}^{3} } {\left( {\eta R_{2}^{2} } \right)}}} \right. \kern-\nulldelimiterspace} {\left( {\eta R_{2}^{2} } \right)}}$$
$$\overline{p}_{ls/rs}$$: $${{p_{ls/rs} } \mathord{\left/ {\vphantom {{p_{ls/rs} } {P_{s} }}} \right. \kern-\nulldelimiterspace} {P_{s} }}$$
$$\overline{p}_{lz/rz}$$: $${{p_{lz/rz} h_{0} } \mathord{\left/ {\vphantom {{p_{lz/rz} h_{0} } {P_{s} }}} \right. \kern-\nulldelimiterspace} {P_{s} }}$$
$$\overline{p}_{{l\dot{z}/r\dot{z}}}$$: $${{p_{{l\dot{z}/r\dot{z}}} h_{0}^{3} } \mathord{\left/ {\vphantom {{p_{{l\dot{z}/r\dot{z}}} h_{0}^{3} } {\left( {\eta R_{2}^{2} } \right)}}} \right. \kern-\nulldelimiterspace} {\left( {\eta R_{2}^{2} } \right)}}$$

## References

[1] Wang Z, Feng X, Du F (2019). A novel method for smooth low-speed operation of linear feed systems. Precis. Eng..

[2] Du F, Zhang M, Wang Z (2018). Identification and compensation of friction for a novel two-axis differential micro-feed system. Mech. Syst. Signal Process..

[3] Yu H, Feng X (2016). Dynamic modeling and spectrum analysis of macro-macro dual driven system. Jilin Water Resources.

[4] Du F, Li P, Wang Z (2017). Modeling, identification and analysis of a novel two-axis differential micro-feed system. Precis. Eng..

[5] Bassani R (1979). The self-regulated hydrostatic screw and nut. Tribol. Int..

[6] Kami Y, Yabuya M, Shimizu T (1995). Research and development of an ultraprecision positioning system. Nanotechnology.

[7] Robert S, Jochen S (2009). Hydrostatic lead screw in compare to linear motor and ball screw. Maschinen Markt (in Chinese).

[8] El-Sayed HR, Khatan H (1975). A suggested new profile for externally pressurized power screws. Wear.

[9] El-Sayed HR, Khataan HA (1976). Study of performance of power screw-nut systems. Wear.

[10] Zhang Y, Lu C, Pan W (2016). Averaging effect on pitch errors in hydrostatic lead screws with continuous helical recesses. J. Tribol..

[11] Zhang Y, Chen S, Lu C (2016). Performance analysis of capillary-compensated hydrostatic lead screws with discontinuous helical recesses including influence of pitch errors in Nut. Tribol.. Trans..

[12] Zhang Y, Lu C, Ma J (2016). Research on two methods for improving the axial static and dynamic characteristics of hydrostatic lead screws. Tribol. Int..

[13] Bair S, Mccabe C (2004). A study of mechanical shear bands in liquids at high pressure. Tribol. Int..

[14] Neto C, Evans DR, Bonaccurso E (2005). Boundary slip in Newtonian liquids: a review of experimental studies. Rep. Prog. Phys..

[15] Vinogradova OI (1999). Slippage of water over hydrophobic surfaces. Int. J. Miner. Process..

[16] Huang P (1999). Theoretical study on the lubrication failure for the lubricants with a limiting shear stress. Tribol. Int..

[17] Chen D, Zhou S, Dong L (2015). Performance evaluation and comparative analysis of hydrostatic spindle affect by the oil film slip. J. Manuf. Process..

[18] Aurelian F, Patrick M, Mohamed H (2011). Wall slip effects in (elasto) hydrodynamic journal bearings. Tribol. Int..

[19] Zhao Y, Wong PL, Guo L (2020). Linear complementarity solution of 2D boundary slip EHL contact. Tribol. Int..

[20] Feng X, Liu Y, *et al*. A linear feed unit with integrated hydrostatic nut main drive screw pair. China: CN112077638A, 2020.

[21] Elsayed HR, Khatan H (1976). The exact performance of externally pressurized power screws. Wear.

[22] Liu JL, Feng XQ, Yu SW (2006). Morphology of liquid drops and thin films on a solid surface with sinusoidal microstructures. Acta. Mech. Sin..

[23] Lauga E, Stone HA (2003). Effective slip in pressure-driven Stokes flow. J. Fluid Mech..

[24] Jian LI, Zhou M, Cai L (2009). On the measurement of slip length for liquid flow over super-hydrophobic surface. Chin. Sci. Bull..

[25] Liang P, Lu C, Ding J (2013). A method for measuring the hydrodynamic effect on the bearing land. Tribol. Int..

[26] Zhang Y, Wang W, He L (2018). Layered oil slip model for investigation of film thickness behaviours at high speed conditions. Tribol. Int..

